# Predicting age at onset of type 1 diabetes in children using regression, artificial neural network and Random Forest: A case study in Saudi Arabia

**DOI:** 10.1371/journal.pone.0264118

**Published:** 2022-02-28

**Authors:** Ahood Alazwari, Mali Abdollahian, Laleh Tafakori, Alice Johnstone, Rahma A. Alshumrani, Manal T. Alhelal, Abdulhameed Y. Alsaheel, Eman S. Almoosa, Aseel R. Alkhaldi

**Affiliations:** 1 School of Science, RMIT University, Melbourne, Victoria, Australia; 2 School of Science, Al-Baha University, Moundq, Saudi Arabia; 3 Pediatric Endocrine Department, Al Aziziyah Maternal and Children Hospital, Jeddah, Saudi Arabia; 4 Pediatric Endocrine Department, Maternal and Children Hospital, Al-Ahsa, Saudi Arabia; 5 Pediatric Endocrine Department, King Fahad Medical City (KFMC), Riyadh, Saudi Arabia; Universiti Malaysia Pahang, MALAYSIA

## Abstract

The rising incidence of type 1 diabetes (T1D) among children is an increasing concern globally. A reliable estimate of the age at onset of T1D in children would facilitate intervention plans for medical practitioners to reduce the problems with delayed diagnosis of T1D. This paper has utilised Multiple Linear Regression (MLR), Artificial Neural Network (ANN) and Random Forest (RF) to model and predict the age at onset of T1D in children in Saudi Arabia (S.A.) which is ranked as the 7th for the highest number of T1D and 5th in the world for the incidence rate of T1D. De-identified data between (2010-2020) from three cities in S.A. were used to model and predict the age at onset of T1D. The best subset model selection criteria, coefficient of determination, and diagnostic tests were deployed to select the most significant variables. The efficacy of models for predicting the age at onset was assessed using multi-prediction accuracy measures. The average age at onset of T1D is 6.2 years and the most common age group for onset is (5-9) years. Most of the children in the sample (68%) are from urban areas of S.A., 75% were delivered after a full term pregnancy length and 31% were delivered through a cesarean section. The models of best fit were the MLR and RF models with *R*^2^ = (0.85 and 0.95), the root mean square error = (0.25 and 0.15) and mean absolute error = (0.19 and 0.11) respectively for logarithm of age at onset. This study for the first time has utilised MLR, ANN and RF models to predict the age at onset of T1D in children in S.A. These models can effectively aid health care providers to monitor and create intervention strategies to reduce the impact of T1D in children in S.A.

## Introduction

Type 1 diabetes (T1D) is a metabolic disorder generally recognised as a result of an autoimmune response that affects insulin-producing *β* cells in the pancreas, which results in extreme insulin deficiencies and associated hyperglycemia [1]. Poor glycemic control as a result of T1D complications can result in diabetic ketoacidosis which may result in significant neurological complications, hospitalization and death [2, 3]. T1D can cause long-term complications like blindness from retinopathy [3] and can also cause kidney failure [4]. In addition, chronic diseases such as diabetes can disturb physiology, impacting linear growth and pubertal development [5]. T1D can cause severe dermatological complications [6]. The incidence rate of T1D worldwide increases by about 3% to 4% per year [7]. Recently, the International Diabetes Federation (IDF) Atlas’ 9th edition (2019), estimated that the number of children and adolescents under the age of 15 years worldwide who are living with T1D was 600,900 [8]. Moreover, it is estimated that more than 98,000 children and adolescents under the age of 15 years are diagnosed with T1D annually, and that number increases to 128,900 when the age range is extended to 20 years [8]. According to the IDF (2019), Saudi Arabia has a high incidence rate of new cases of T1D in children and adolescents (<15 years of age) at 31.4 cases/100,000 children each year, which places it as the 5th highest worldwide [8]. The 9th IDF editions reported the number of new cases of T1D among children under 15 years to be 2,800 [8].

This highlights the urgent need for an improved understanding of the development of T1D in Saudi Arabian children to facilitate improved monitoring to reduce the complications of delayed diagnosis. In addition, this may assist the practicality of early intervention trials, to increase the chance of disease mitigation prior to the onset of dysglycemia to retain a greater number of functional islet cells.

### Literature survey

The onset of T1D is affected by multiple genetic and environmental risk factors [9–12]. The roles of the environment and genetics on the development of T1D have been recognised for more than 40 years [13], however, determining the environmental and perinatal risk factors of T1D is ongoing [10, 11, 14]. Risk factors shown to be associated with T1D onset include childhood infections, diet, family history of diabetes [10, 11, 15] and perinatal factors [16] while the relative contribution of each factor has not been clearly determined [10]. Published studies related to modelling age at onset of T1D were reviewed and the issues in the context of the proposed paper are summarised in S2 Table. It has been reported that preterm (<37 weeks) [17], and birth weight [17, 18] were associated with earlier onset of T1D in children, however, were not significant risk factors for developing T1D [19]. Early T1D onset in children was related to family history of T1D [20, 21], in siblings [22], or in parents [19], but maternal diabetes was significantly associated with an older age of onset [22]. Maternal age of 25–29 years, or the father’s age of ≥ 30 years were both identified as risk factors of early T1D in children in [19] whereas they were not found to be significant risk factors where paternal age greater than 25 years was considered by [20] nor with maternal age at delivery (≥35 years) [19]. A study conducted in the UK [23], reported that in consanguineous pedigrees, Wolcott-Rallison syndrome is the most common cause of chronic neonatal diabetes. Also, children who were younger at the time of diagnosis tended to be heavier [24–26], and taller [24, 26]. Other risk factors such as season of birth, year of birth, gestational age size [17], mixed feeding, children’s prior history of infections [21], cesarean section, gestational diabetes, pre-eclampsia [19] and maternal weight at childbirth [27] were linked to early onset of T1D in children. Gender, ethnicity, and a history of autoimmune disease in the family [17, 21], higher birth order, multiple bacterial infections, and residing in high population density areas were not associated with early onset of T1D [19]. In addition, studies from Saudi Arabia that have examined factors contributing to T1D in children have indicated that the incidence of childhood T1D may be associated with vitamin D deficiency [28, 29]. Therefore, the possibility to prevent, delay or reduce complications of T1D diabetes in children is an important area of research [3, 30, 31]. In the preventative studies [32, 33], it was shown that elimination of cow’s milk proteins in infant formula (in the Finish TRIGR pilot research [32]) or the elimination of bovine insulin in infant formula (in the FINDIA study [33]) both reduced the production of islet autoantibodies. However, the existing T1D research such as those conducted in Sweden and Finland [34, 35] do not represent the ethnicity and diversity of the Saudi Arabia population. This study aims to fill the gap by developing predictive models to estimate the age at onset of TID using data from Saudi Arabia.

Further studies have investigated methods for prediction of age at onset of T1D. The early exposure to respiratory infections was shown to have a higher risk for autoantibody seroconversion in children with a family history of T1D [36]. This was identified through on going monitoring islet autoantibodies during their first 3 years of life [36]. Longitudinal autoantibody measurements have also been used as a risk predictor in families that have a first-degree relative with T1D [37], in the general populations [38] or in individuals identified as being at risk [37, 39, 40]. Also, genetic factors and genetic risk scores were used to identify the presence of islet autoantibodies in children with high-risk HLA genotypes [41–44]. Examining metabolic changes indicates that post-challenge C-peptide levels start to drop significantly six months before diagnosis [45]. A combined risk score model including clinical, genetic, and immunological characteristics created for high-risk children (who were followed from birth until 9 years) showed a significantly improved T1D prediction compared to autoantibodies alone [46]. However, beyond the above research there is a lack of application of machine learning methods for developing models of age at onset of T1D. This is despite many studies utilising various methods of machine learning for type 2 diabetes [47–49]. Hence, the proposed work differs from previous research in that it models the age at onset of T1D in children by using statistical and machine learning models to identify the risk factors and create a predictive model.

### Motivation and the objective of the proposed research

Saudi Arabia has an increasing incidence rate of T1D in children and it is ranked as the 7th for the highest number of T1D and 5th in the world for the incidence rate of T1D. Despite the remarkable increase in the incidence of childhood T1D in Saudi Arabia, there is a lack of meticulously carried out research on T1D in children in Saudi Arabia compared with developed countries [50, 51]. In addition, most of the published research of TID in children in Saudi Arabia are cross-sectional with small sample sizes and involve a single center and a single city/region of the country [50]. Consequently, prior studies do not accurately represent the country’s large and diverse population. Hence, it is important to carry out research on modeling the age at onset of T1D, with the aim to reduce the problems with delayed diagnosis of T1D in Saudi Arabia. This will both support the improvement of the health of the nation and add to the current research of T1D in diverse populations, while recognising the lack of T1D studies for Saudi Arabia children. The existing T1D research conducted in Saudi Arabia have examined the different aspects of T1D in children but not have modelled the age at onset of T1D in children. This study aims to fill the gap by utilising a secondary data source, curated specifically for this study to develop the most suitable predictive model for predicting age at onset of T1D in children. As to the best of our knowledge, no previous studies have modelled the age at onset of T1D in children in Saudi Arabia and identified the risk factors. In addition, there is no study in the literature comparing the following chosen methods of multiple linear regression (MLR), artificial neural network (ANN) and Random Forest (RF) for the age at onset of T1D in children <15 years old using local data.

The results of this study indicate that MLR and RF models outperform the ANN model for age group of <15. However, the RF model performs better than MLR and ANN models for the most common age group (5–9) based on coefficient of determination *R*^2^, root-mean-square error (RMSE) and mean absolute error (MAE).

## Data and methods

This section outlines the data collection, the development and evaluation of the prediction models using multiple linear regression (MLR), artificial neural network (ANN) and Random Forest (RF) methodologies.

### Data collection

**Fig 1:**
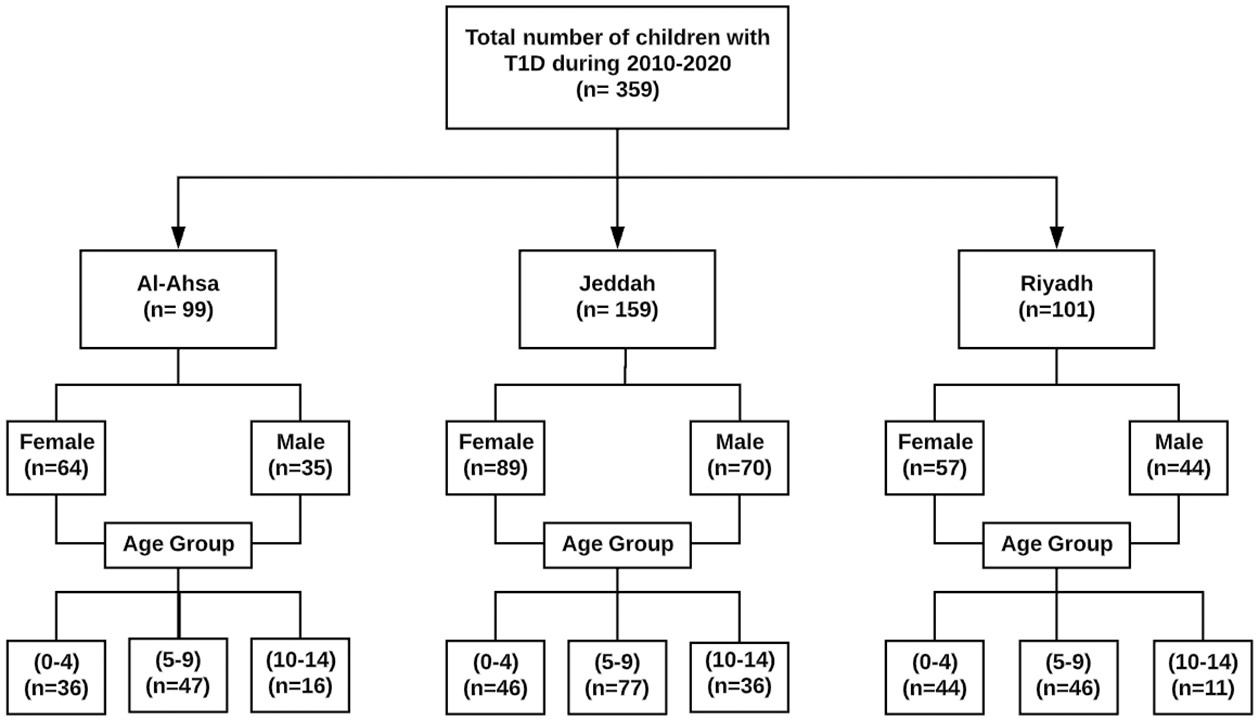
Demographic summary of the cohort of children with T1D collected from three diabetes clinics located in three major cities in Saudi Arabia with diagnosis between 2010 and 2020.

De-identified data for 359 individuals were collected using medical files from three diabetes clinics, located in three major cities of Saudi Arabia. Ethical approval was obtained from the RMIT University Human Research Ethics committee in Australia and the Research Ethics Committee of the Ministry of Health in Saudi Arabia. The need for informed consent was waived by the ethics committee as this was a retrospective study of medical records. All data were fully anonymized before analysis. An overview of the demographic breakdown of the data are given in (Fig 1). Additional demographic data was collected based on gender, residency, consanguineous parents, birth weight, birth year, and birth order, pregnancy factors such as gestational age in weeks and mode of delivery (normal delivery/caesarean section), clinical history such as family history of diabetes, child’s weight and height and maternal characteristics such as maternal age at child’s birth. Pregnancy length was grouped based on the gestational weeks used by the World Health Organisation (WHO) and American College of Obstetricians and Gynaecologists Committee on Obstetric Practice Society for Maternal-Foetal Medicine [52, 53], ≤36 (preterm), 37–38 (early term), 39–40 (full term), 41 (late term) and ≥42 (post term).

### Model development

This study compares the full cohort in addition to a focused study on the common age group for T1D diagnosis of (5–9) years to model the age at onset of T1D.

As previously shown in the literature [17–26], the child’s gender, having a family history of T1D, pregnancy length, birth year, birth weight, residency, mode of birth delivery, consanguineous parents, maternal age at birth, birth order, elevated weight at diagnosis and height at diagnosis could influence the age at onset of T1D and were included in the analysis. The efficacy of models were assessed using coefficient of determination *R*^2^, root-mean-square error (RMSE) and mean absolute error (MAE).

MLR, ANN and RF models have been used in many studies to describe different systems [54–61], and hence were chosen to model the age at onset of T1D in this cohort. The statistical software R was used to perform the analysis [62].

#### Multiple linear regression (MLR)

MLR [63] is used in this study as a prediction technique to model age at onset of T1D based on the independent variables suggested from the literature and additional variables collected in this study. The general MLR model is defined by the following equation:

$$\begin{array}{c} y=\beta_{0}+\beta_{1}x_{1}+\beta_{2}x_{2}\cdots \beta_{k}x_{k}+\varepsilon , \end{array}$$ (1)

where y is the dependent variable (age at onset), *β*_0_ is the intercept, *β*_1_, ⋯, *β*_*k*_ are the regression coefficients for the independent variables or interaction terms, *x*_1_, ⋯, *x*_*k*_ are independent variables and *ε* is the residual term of the model.

#### Artificial Neural Network (ANN)

**Fig 2:**
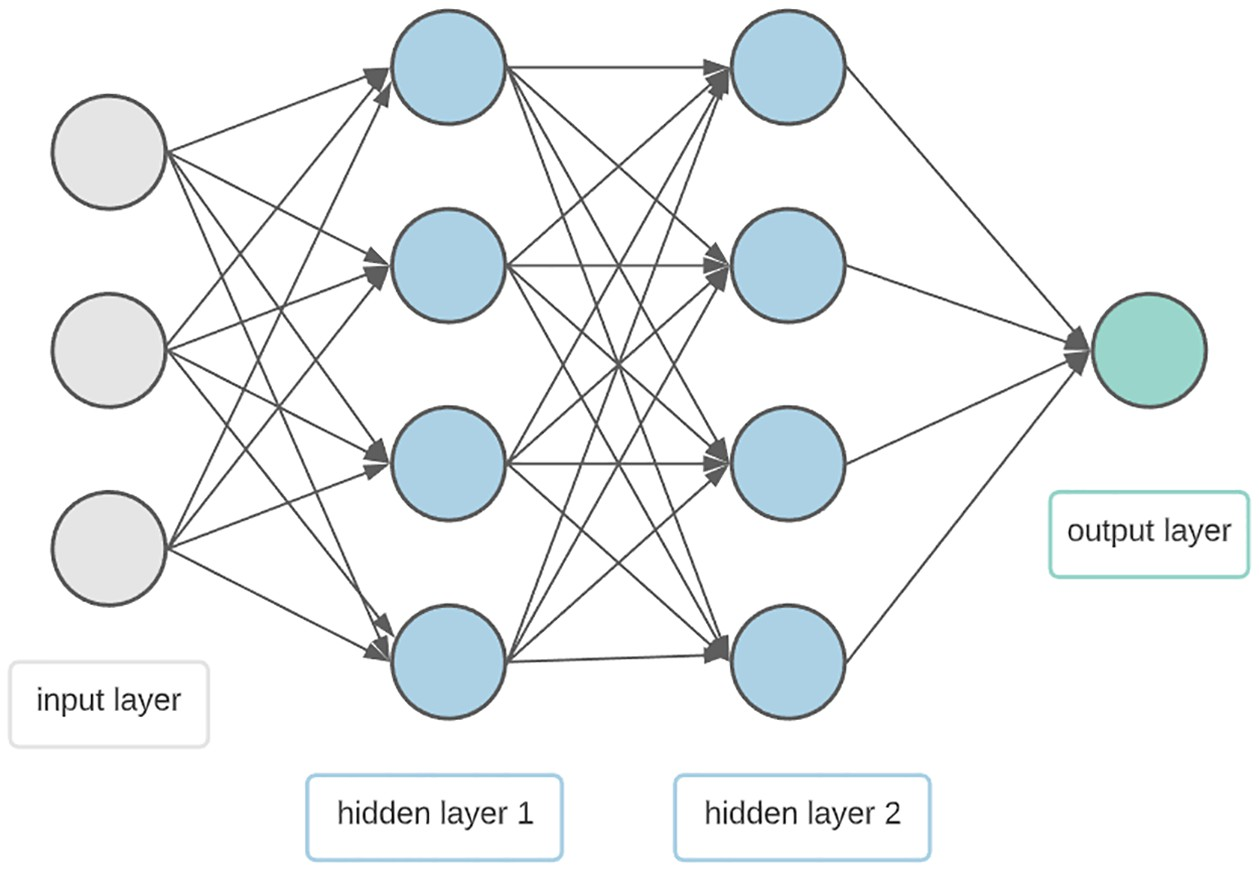
Artificial neural network with 3 inputs and two hidden layers having four hidden neurons and one output.

Advances in machine learning in the medical area have recently provided new opportunities in the field of disease prediction and prescription treatment [64]. ANN is a branch of the wider field of machine learning. It is one of the well known prediction approaches used for finding a solution when other statistical methods can not be effectively utilized. The benefits of this method, including the ability to learn from instances, fault tolerance and non-linear data forecasting, make it a suitable statistical method [65]. One of the major benefits of ANN is its ability to distinguish hidden linear and nonlinear relationships, often in high-dimensional and complex data sets [66]. ANN consists of input, hidden and output layers known as neurons [67] (Fig 2). The number of input layer neurons represents the number of variables which describe the features being evaluated, whereas the output layer neuron is the dependant variable. The number of hidden layers and the number of neurons depend on the quantity of data and the complexity of the relationship between the input and output layers. Every neuron in the hidden and output layer are linked by a corresponding numerical weight to all neurons in the proceeding layer [68].

#### Random Forest (RF)

Decision trees have become a very common machine learning tool in recent years due to its simplicity, ease of use and interpretability [69]. Various studies have been performed to address the limitations of conventional decision trees such as lack of robustness and suboptimal performance [70]. The development of an ensemble of trees followed by a vote of the most common class, is one of the most useful techniques that resulted from these studies [71]. Random forest (RF) is an ensemble learning approach and the output of a number of weak learners which may be a single decision tree is improved through a voting scheme similar to other ensemble learning methods [54]. Since RF has a built-in feature selection method, it can handle a large number of input variables without the need to minimize dimensionality and the overfitting can be controlled by using out-of-bag validation [72].

## Results

### Descriptive statistical analyses of cohort of T1D in Saudi Arabia

**Fig 3:**
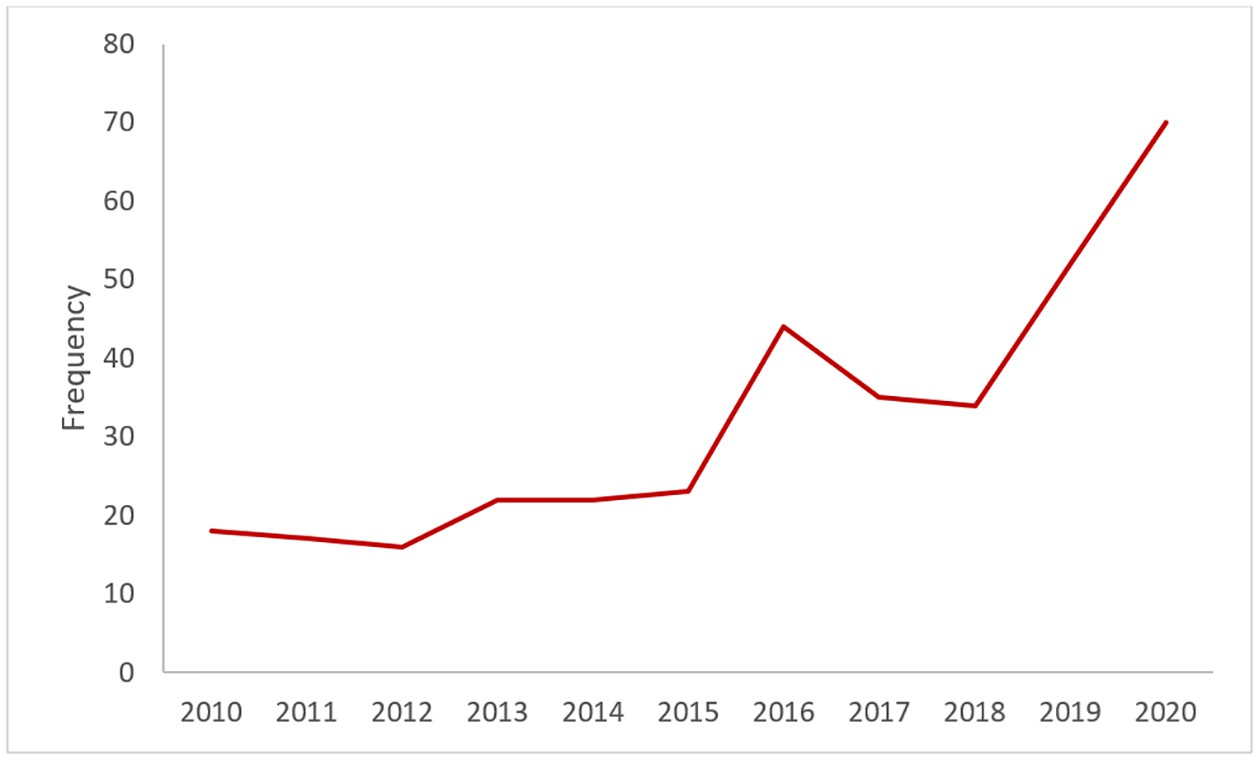
Cases of childhood T1D between 2010 and 2020 in the three diabetes centers of Saudi Arabia used in this study.

**Fig 4:**
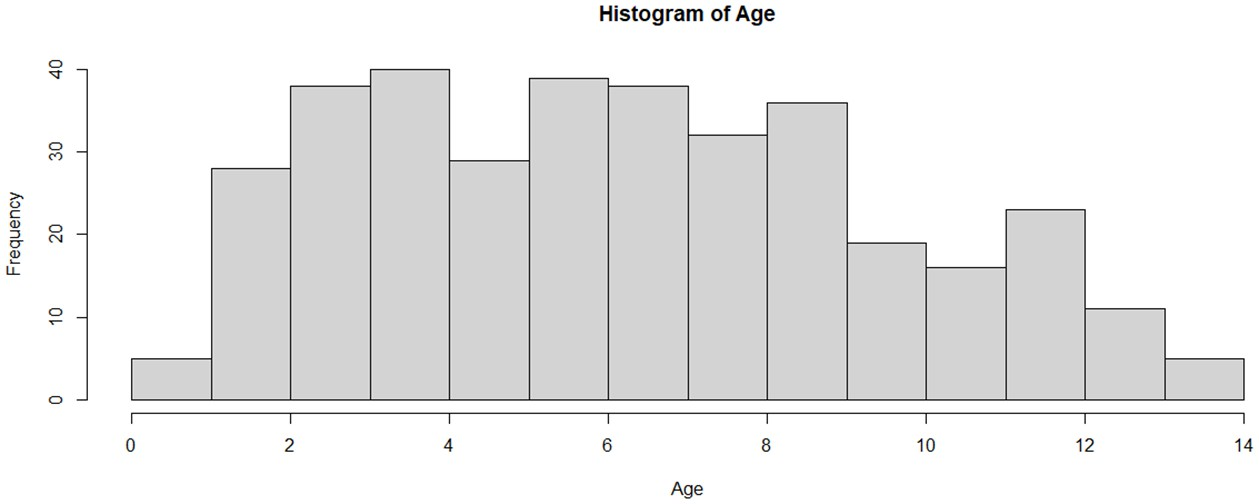
Distribution of age at onset of cohort collected from three cities in Saudi Arabia: Al-Ahsa, Jeddah and Riyadh (2010–2020).

**Fig 5:**
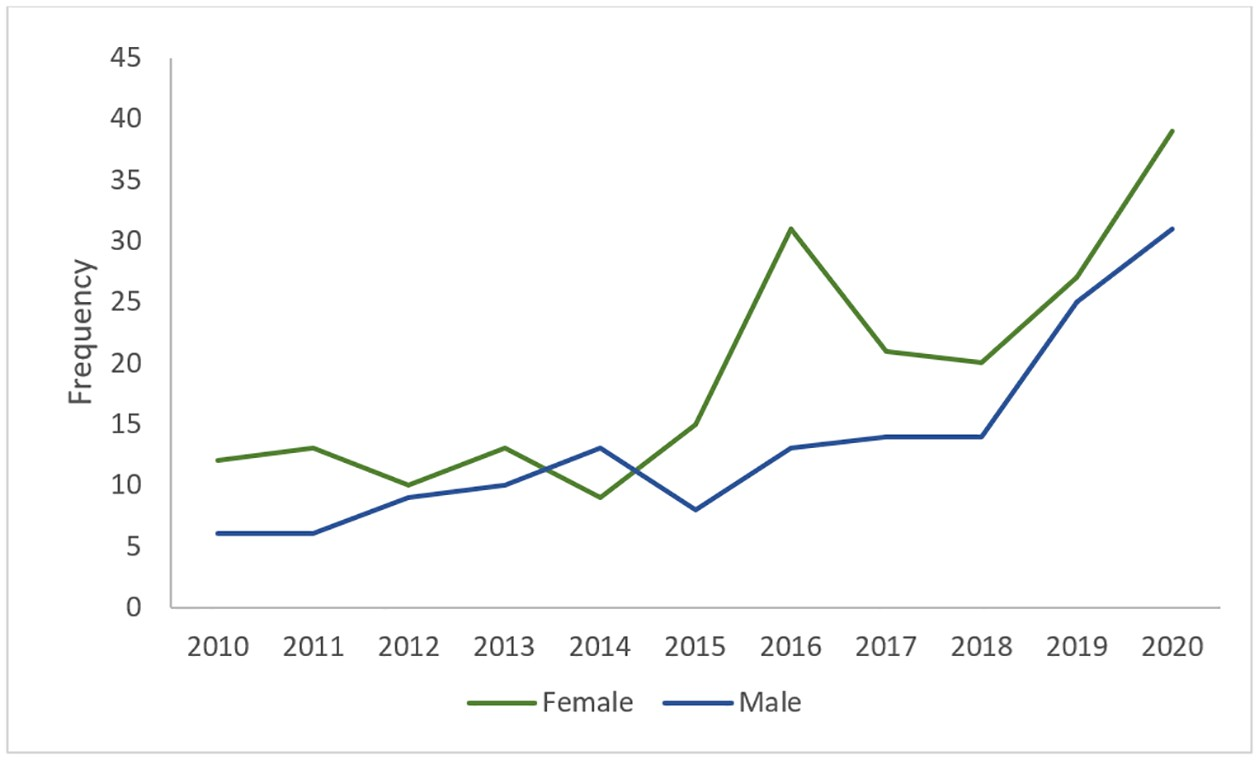
Trend of cases by gender of 359 data during the period 2010 and 2020.

The trend of the reported cases of children with T1D between 2010 and 2020 is shown in (Fig 3). It can be seen that there has been an upward trend in the number of reported cases in these centres during this period. As shown in Table 1, the mean age at onset of T1D in this cohort was 6.2 years with standard deviation of 3.28. For males (n = 149), the mean age at onset was 6.1 years with standard deviation of 3.18 while for females (n = 210) the average is 6.3 years with standard deviation of 3.35. The median and mode for the full cohort were 6 and 8.1 years, respectively. The maximum age of diagnosis was 13.9 years while an age of 1 month was the minimum age (Table 1). Females scored only slightly less than the males on the average age at onset of T1D, but the difference was not large enough to be statistically significant (t = 0.56909, p = .569) (Table 2). In addition, there is a significant interaction between gender and cities that shows a difference for males in Riyadh compared to males in Jeddah (Table 3). The full ANOVA Results of the interaction between gender and cities is provided in S2 Table. Also, as shown in (Fig 4), the distribution of the age at onset of T1D was not normal (Shapiro-Wilk test, p-value<0.001, Kurtosis = -0.808). The mean height of this group was (1.20 ± 0.20) metres, the mean weight was (22.6 ± 11.02) kg and the mean birth weight was (2.90 ± 0.64) kg. The range of birth year for this cohort is from (2003 to 2020). The median birth year was 2010. The number of females in this sample is higher than males with 210 females compared to 149 males. In addition, (Fig 5) illustrates that the females have higher incidence of T1D than males over this period. We can also see an approximately even distribution across sites with 28% of the cases from Al-Ahsa, 44% from Jeddah and 28% from Riyadh. The majority of the sample (68%) are from urban areas of Saudi Arabia.

**Table 1:** Descriptive statistics for age at onset of T1D.

	All	Female	Male
N	359	210	149
Mean	6.2	6.3	6.1
Std.Deviation	3.29	3.35	3.13
Median	6.1	6.0	6.1
Mode	6.1, 8.1	8.1	7.0
Maximum	13.9	13.9	13.9
Minimum	0.1	0.11	0.1

**Table 2:** T-test results comparing age at onset of T1D between males and females.

Gender Level	N	Mean	CI	t	df	p-value
Male	149	6.1	(-0.487211, 0.883844)	0.56909	328.81	0.569
Female	210	6.3				

**Table 3:** ANOVA results comparing age of onset of T1D between gender and cities.

		Mean Difference	CI	P-value
Gender	Male-Female	-0.198316	(-0.889906, 0.493273)	0.573
Cities	Jeddah-Al-Ahsa	0.509462	(-0.473089, 1.492014)	0.441
	Riyadh-Al-Ahsa	-0.516384	(-1.601806, 0.569037)	0.502
	Riyadh-Jeddah	-1.025847	(-2.002385, -0.049308)	0.033*
Gender:City	Male:Riyadh-Male:Jeddah	-2.019123	(-3.818642, -0.219603)	0.017*

Almost half of the cases (47%) have parents that are consanguineous and 49% of all cases have a family history of diabetes. The majority (75%) were delivered after a full term pregnancy length and 31% were delivered through a caesarean section. 32% of cases were the firstborn child. 57% of cases at birth were to mothers aged between 25 and 35 years old.

**Fig 6:**
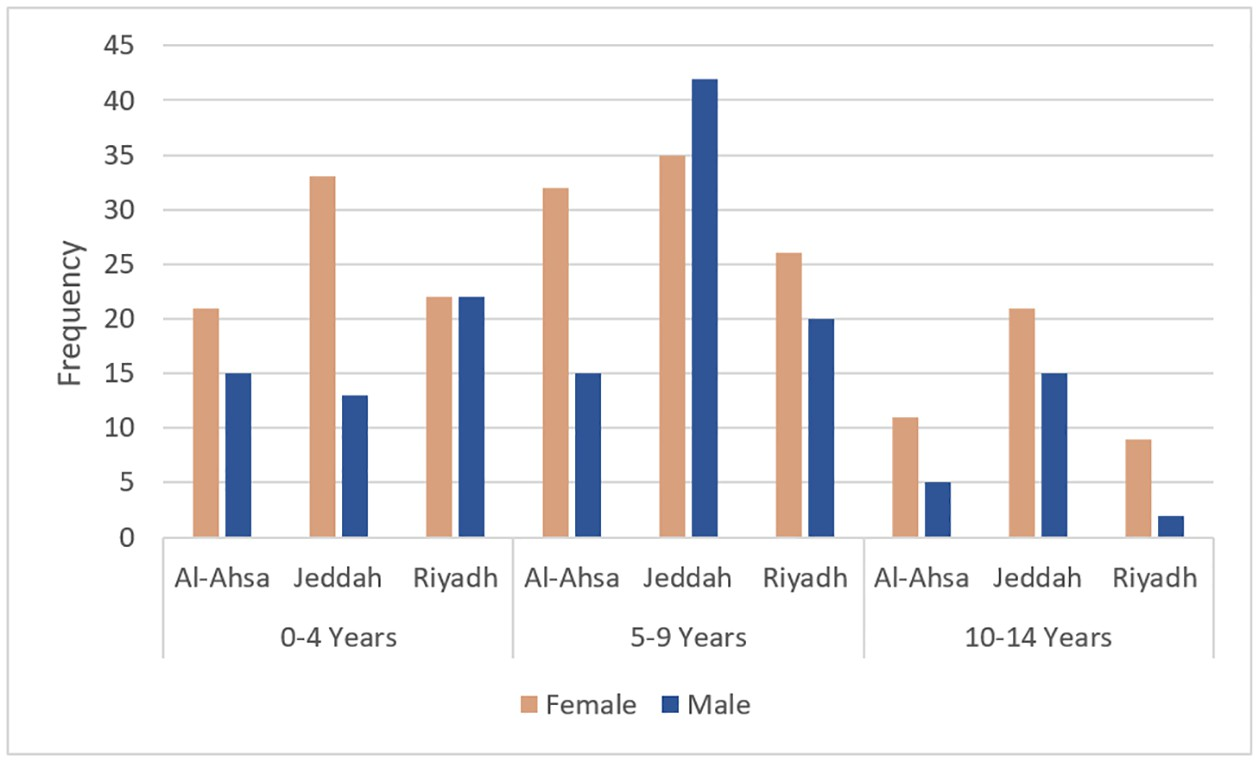
Distribution of gender and age at onset of T1D by cities.

Across the three cities the distribution of age and gender is also different (Fig 6). In Al-Ahsa and Riyadh, the majority of patients are females in all age groups whereas in Jeddah, the number of males were higher than females in the age group 5–9 years. The figure also shows that the most common age group of children for onset of T1D in the three cities is 5–9 years.

### Multiple linear regression modeling (MLR)

We have developed MLR models based on the dependent variable age at onset (y) and also investigated transformations of the dependent variable; the square root of y and the logarithm of y. To improve the efficacy of the MLR models, interactions between independent variables were considered. The MLR models with interactions were selected based on the step-wise selection criteria of the smallest Akaike’s Information Criteria (AIC). Table 4 illustrates the variables in each MLR model. Table 5 shows the results of MLR models together with their corresponding *R*^2^, Adjusted *R*^2^, RMSE and MAE. In Table 5, the best model was MLR model(6), which contains independent variables in addition to interactions between variables shown in Table 10. Comparison of the MLR models in Table 5 shows that the transformation of the dependent variable decreases the values of RMSE and MAE.

**Table 4:** Independent and interaction variables in selected MLR models.

MLR models	Variables
(a) M1:y	birth weight, birth year, birth order, city, C.P., height, P.L., residency, weight, P.L.:residency, birth year:height, birth order:residency, C.P.:height, birth weight:C.P., height:weight, birth weight:height, birth weight:weight, birth weight:birth year, residency:weight, C.P.:residency, C.P.:weight, birth weight:height:weight.
(b) M2:sqrt(y)	birth weight, birth year, birth order, city, C.P., B.D.M, height, M.Age, P.L., residency, weight, P.L.:residency, birth order:city, height:weight, birth weight:height, height:M.Age, birth weight:C.P., C.P.:height, city:weight, B.D.M.:M.Age, birth weight:residency, birth order:B.D.M., B.D.M:residency, birth year:B.D.M.
(c) M3:log(y)	birth weight, birth year, birth order, city, C.P., F.H., height, M.Age, P.L., residency, weight, height:weight, birth order:city, P.L.:residency, birth weight:height, birth weight:weight, height:M.Age, C.P.:height, birth weight:C.P., birth year:city, city:F.H., birth weight:height:weight.

C.P.:Consanguineous Parents, F.H.: Having a family history of T1D, P.L.: pregnancy length, B.D.M.: Birth delivery mode, M.Age: Maternal age at child’s birth, (:): interactions between variables.

**Table 5:** MLR models of age at onset of T1D.

Models without interactions	*R* ^2^	Adj *R*^2^	RMSE	MAE
M1:lm(y, x’s)	0.80	0.79	1.45	1.13
M2:lm(sqrt(y), x’s)	0.80	0.80	0.32	0.25
M3:lm(log(y), x’s)	0.70	0.68	0.40	0.27
Models with interactions	*R* ^2^	Adj *R*^2^	RMSE	MAE
M4:lm(y, x’s)	0.86	0.84	1.25	0.97
M5:lm(sqrt(y), x’s)	0.85	0.83	0.27	0.21
M6:lm(log(y), x’s)	0.89	0.86	0.24	0.18

(Fig 7) shows the observed values versus fitted values for the MLR models. The plot shows that the MLR model (6) of the logarithm of age at onset of T1D with interactions between variables (c2) was the best model based on the value of *R*^2^, adjusted *R*^2^ and the smallest values of RMSE and MAE.

**Fig 7:**
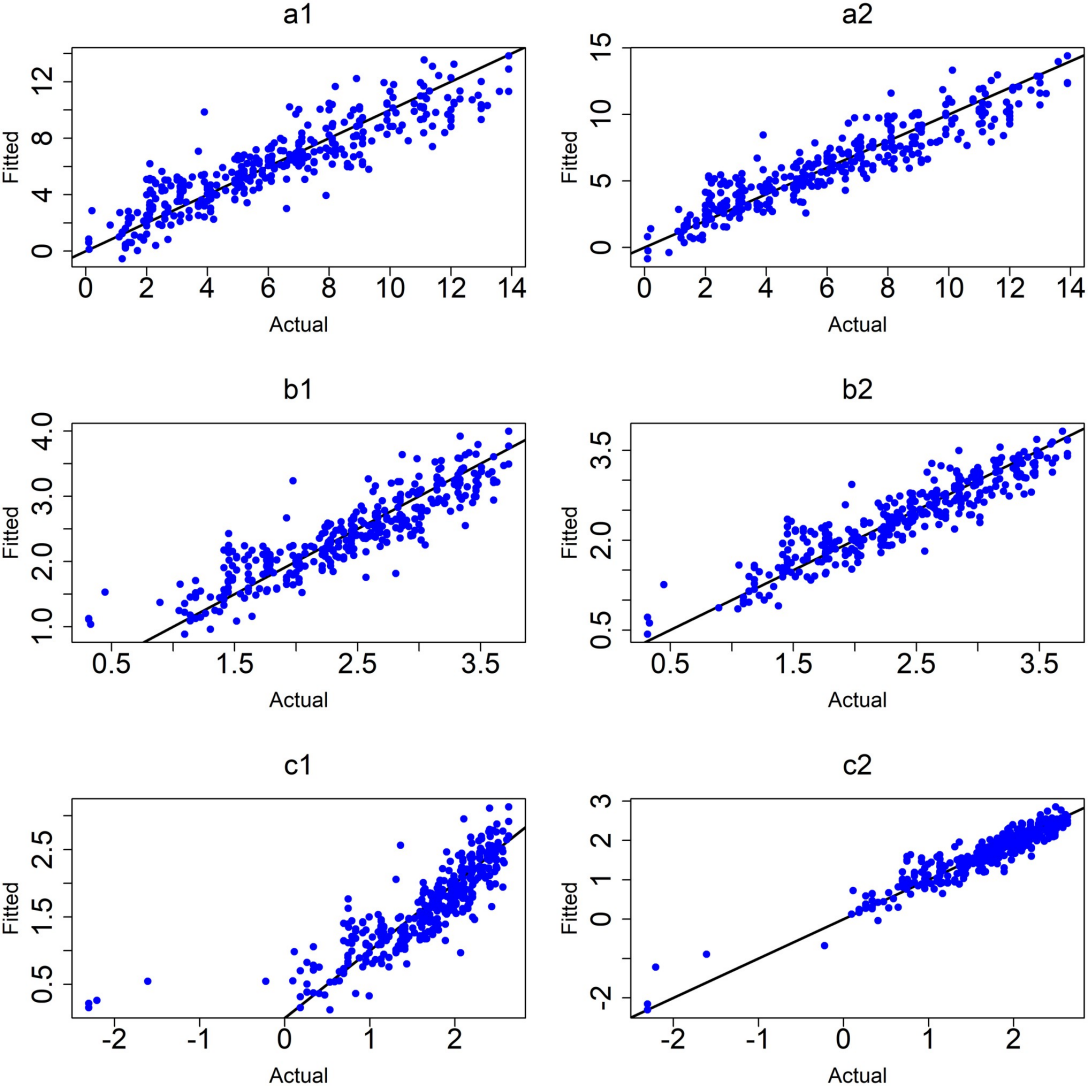
MLR models. (a1) M1:y without interactions and (a2) M4:y with interactions, (b1) M2:sqrt(y) without interactions and (b2) M5:sqrt(y) with interactions and (c1) M3:log(y) without interactions and (c2) M6:log(y)with interactions.

**Fig 8:**
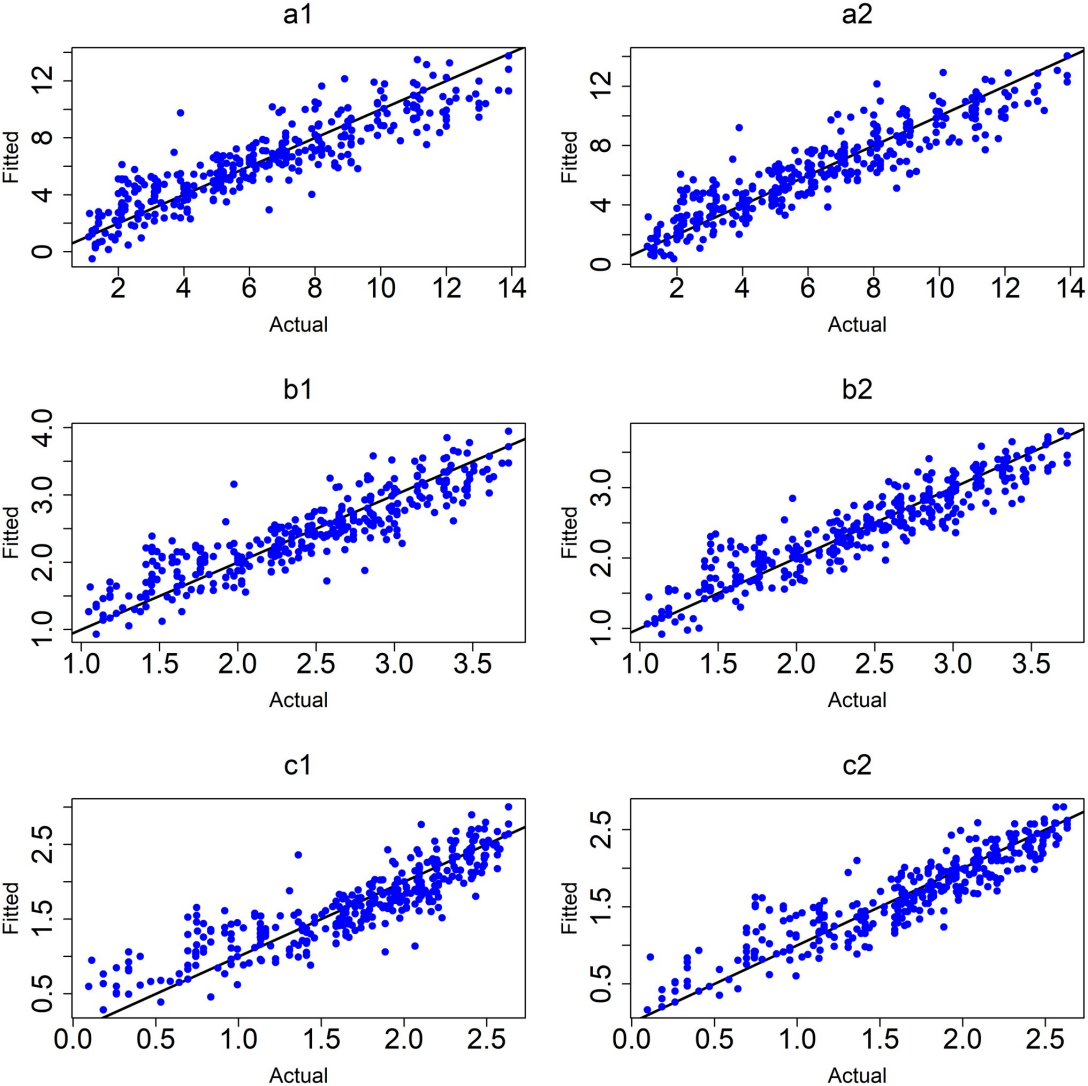
MLR models after removing outliers. (a1) M1:y without interactions and (a2) M4:y with interactions, (b1) M2:sqrt(y) without interactions and (b2) M5:sqrt(y) with interactions and (c1) M3:log(y) without interactions and (c2) M6:log(y)with interactions.

To further improve the MLR models and address potentially influencing outliers, the cases that had age at onset of T1D less than one year were removed from further analysis. The results in Table 6 and (Fig 8), indicated that the best model was still M6 the logarithm of age at onset of T1D which achieves *R*^2^ of 0.85 and the smallest values of RMSE and MAE (0.25 and 0.19).

**Table 6:** MLR models of age at onset of T1D after removing outliers (N = 354).

Models without interactions	*R* ^2^	Adj *R*^2^	RMSE	MAE
M1:lm(y, x’s)	0.80	0.79	1.45	1.13
M2:lm(sqrt(y), x’s)	0.80	0.79	0.30	0.24
M3:lm(log(y), x’s)	0.77	0.76	0.29	0.23
Models with interactions	*R* ^2^	Adj *R*^2^	RMSE	MAE
M4:lm(y, x’s)	0.83	0.82	1.32	1.02
M5:lm(sqrt(y), x’s)	0.85	0.83	0.26	0.20
M6:lm(log(y), x’s)	0.85	0.83	0.25	0.19

(Fig 8) shows the observed values versus fitted values for the MLR models without and with interactions after removing outliers.

### Artificial Neural Network modeling (ANN)

**Fig 9:**
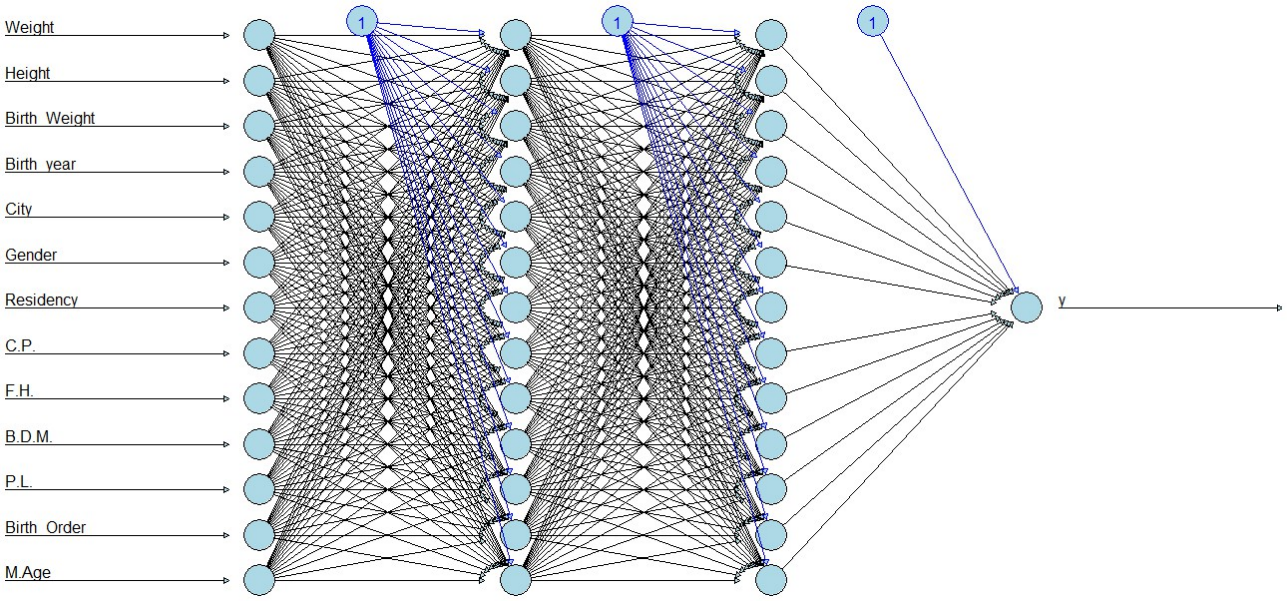
ANN diagram. The weights between neurons is represented by the lines, the black lines show positive relationships and grey lines show negative relationships. Line thickness represents weights’ values.

Data were randomly divided into two subsets of 80% for training to build the ANN models and 20% to use as a testing set to assess the validity of the ANN. For ANN, there is no general rule for determining the number of neurons in the hidden layers [73]. The number of hidden layer neurons varies from problem to problem and it depends on the number and quality of training patterns [74]. The chosen model has an input layer with 13 inputs, the two hidden layers have 13 neurons and the output layer has one output. All independent variables (X’s) were used as input data for the ANN. To assess the effect of the hidden layers on neural network output, the number of neurons in a hidden layer was varied. Higher number of neurons did not make a significant difference in the performance of the ANN models, so 13 neurons have been chosen to reduce the complexity of network (Fig 9). Table 7 shows the results of ANN models.

**Table 7:** ANN models of age at onset of T1D.

Models	Training data			Testing data		
	*R* ^2^	RMSE	MAE	*R* ^2^	RMSE	MAE
A1: y	0.70	2.25	1.84	0.77	2.53	2.06
A2:sqrt(y)	0.64	0.55	0.48	0.72	0.59	0.51
A3:log(y)	0.67	0.50	0.43	0.73	0.52	0.44

**Fig 10:**
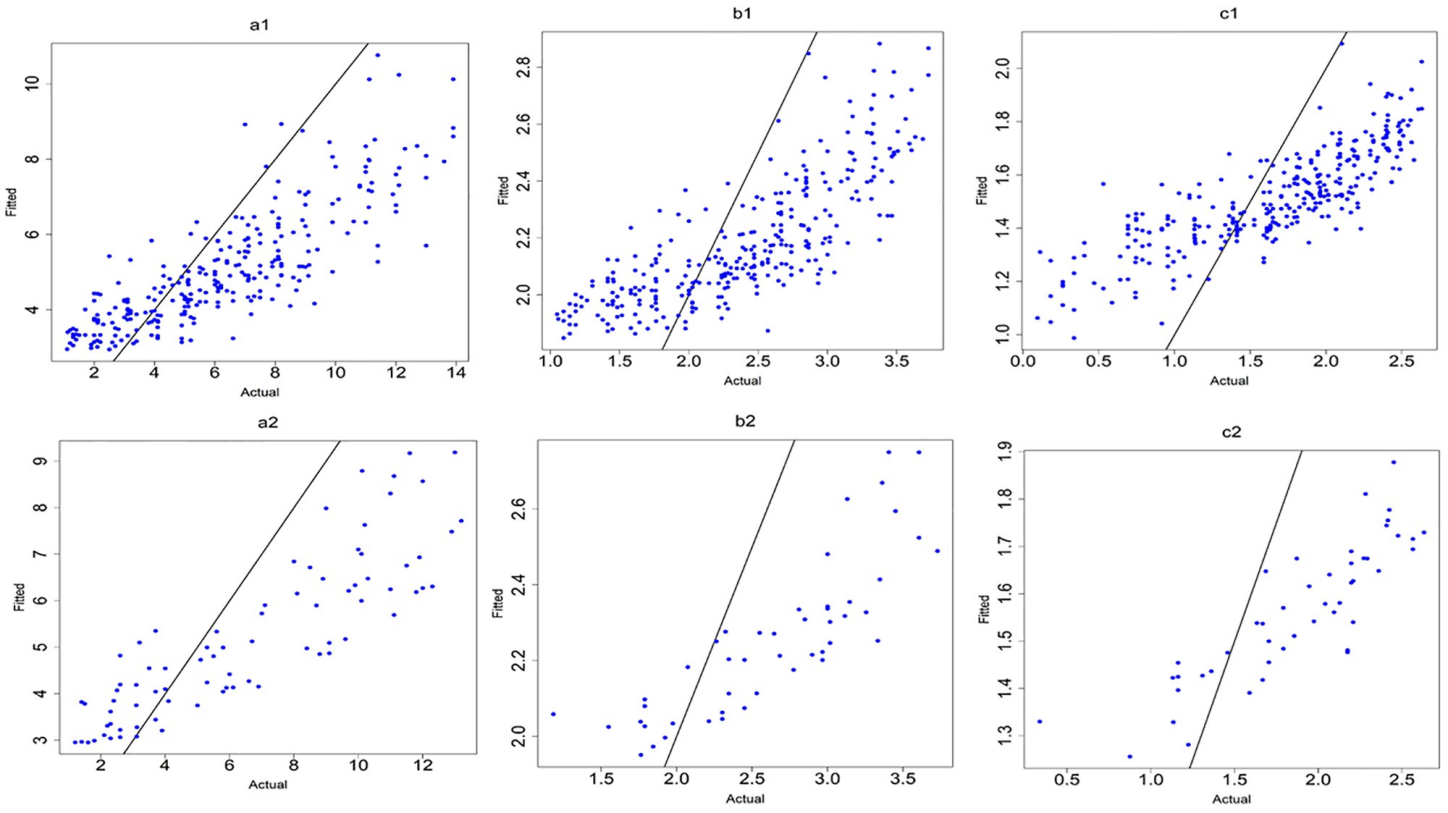
Plots of testing data of ANN models after removing outliers. (a1,a2) y: age at onset, (b1,b2) y: the square root of age at onset, (c1,c2) y: the logarithm of age at onset.

Comparing the results for the testing data, the ANN models for age at onset, square root of age at onset and logarithm of age at onset have *R*^2^ of 0.77, 0.72 and 0.73, RMSE of (2.53, 0.59 and 0.52) and MAE (2.06, 0.51 and 0.44) respectively as shown in Table 7. Therefore, based on *R*^2^, the ANN model A1 of age at onset of T1D outperformed the other ANN models.

(Fig 10) shows the plots of observed values versus predicted values of the ANN models for the training and testing data.

### Random Forest modeling (RF)

Data were randomly divided into two subsets consisting of 80% for training to build the RF models and 20% to use as a testing set to assess the validity of models. In the RF models, the number of trees was set at 500 and the number of variables in each node was set at 4. The results of RF models are shown in Table 8. The RF models for age at onset, square root of age at onset and logarithm of age at onset all have *R*^2^=0.95 for the training data while the testing data had *R*^2^ of (0.87, 0.89 and 0.89) respectively. As the square root of age at onset and logarithm of age at onset models both have the higher *R*^2^ of 0.89, the logarithm of age at onset was therefore chosen as the best RF model (RF3) based on the smaller RMSE and MAE.

**Table 8:** Random forest models of age at onset of T1D.

Models	Training data			Testing data		
	*R* ^2^	RMSE	MAE	*R* ^2^	RMSE	MAE
RF1: y	0.95	0.75	0.57	0.87	1.26	0.94
RF2:sqrt(y)	0.95	0.16	0.12	0.89	0.24	0.19
RF3:log(y)	0.95	0.15	0.11	0.89	0.21	0.17

(Fig 11) shows the plots of observed values versus predicted values of the RF models for the training and testing data.

**Fig 11:**
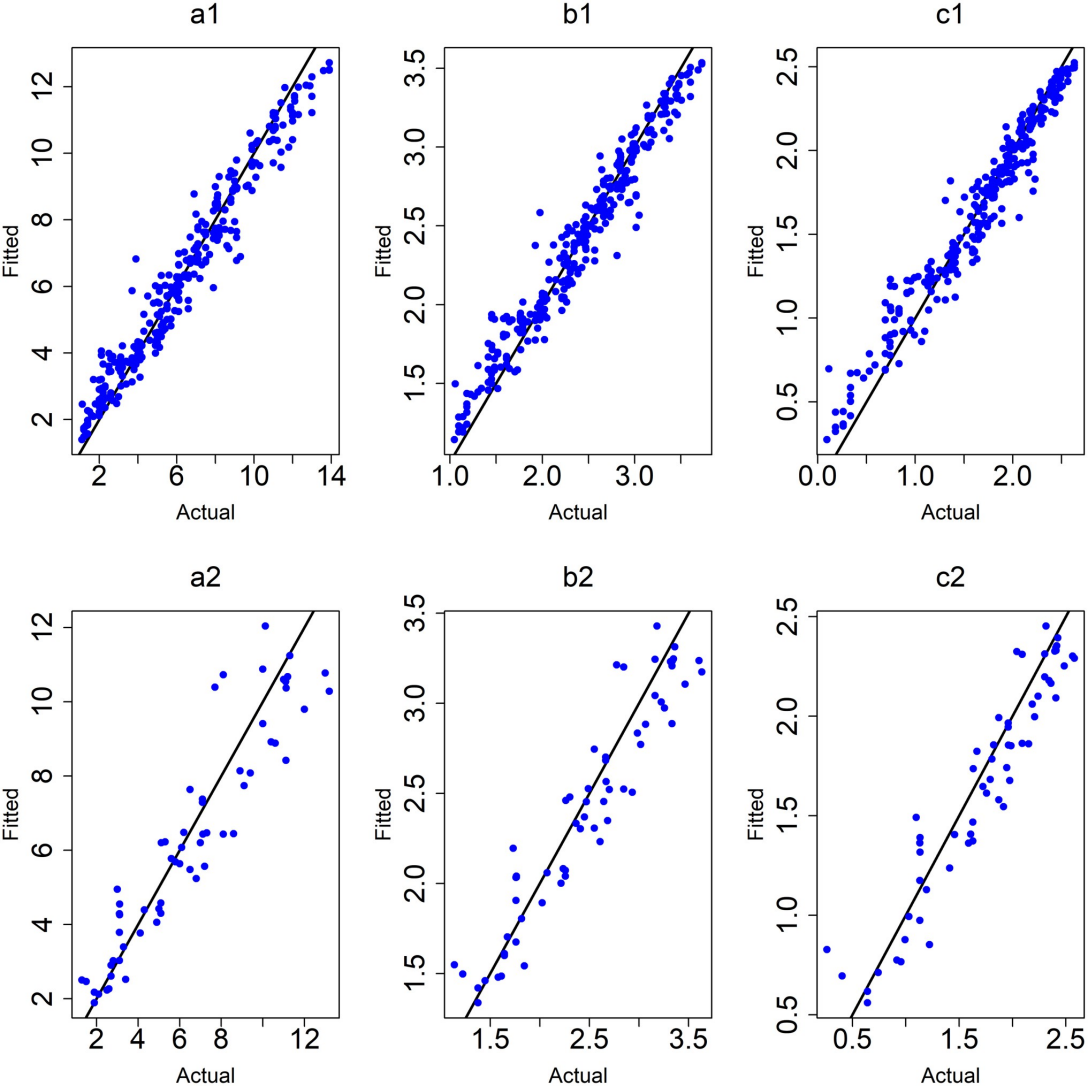
Plots of training and testing data of RF models after removing outliers. (a1,a2) y: age at onset, (b1,b2) y: the square root of age at onset, (c1,c2) y: the logarithm of age at onset.

## Model validation

Validation of the best model of each MLR, ANN and RF was conducted based on their corresponding, *R*^2^, RMSE and MAE when applied to the test data set. The results are summarized in Table 9 and clearly show that MLR and RF outperform the ANN model with *R*^2^ = 0.88 and 0.89, RMSE = (0.22 and 0.21) and MAE = (0.18 and 0.17) respectively. In addition, the selected MLR and RF models both show a high accuracy of 0.99.

**Table 9:** Comparison between MLR,ANN and RF models of age at onset of T1D with test data N = 61.

Models	*R* ^2^	RMSE	MAE	Accuracy
M6:(log(y), X’s)	0.88	0.22	0.18	0.99
A1:(y, X’s)	0.77	2.53	2.06	0.92
RF3:(log(y), X’s)	0.89	0.21	0.17	0.99

## Age at onset of T1D, considering environmental factors and family history of diabetes

To assess the impact of environmental factors on the age at onset of T1D the selected MLR, ANN and RF models (M6, A1 and RF3) were utilized.

### Risk factors of age at onset of T1D based on MLR

The analysis of the variables that influence the age at onset of T1D based on the best MLR model with their corresponding P-value and 95% confidence interval is provided for the full model in Table 10 (non significant interaction variables were excluded for brevity).

**Table 10:** Variables of the MLR model.

Variables	*β*	CI	P-value
Intercept	0.000000	(-0.146981, 0.146981)	0.000***
Birth weight	-0.091424	(-0.138910, -0.043938)	0.020*
Birth year	-0.286026	(-0.349947, -0.222104)	0.000**
Birth order:1st	-0.037343	(-0.187205, 0.112517)	0.522
Birth order:2nd	0.034732	(-0.145992, 0.215457)	0.553
Birth order:3rd	0.066655	(-0.088805, 0.222116)	0.203
City(Jeddah)	0.137425	(-0.009907, 0.284757)	0.025*
City(Riyadh)	-0.103959	(-0.271858, 0.063939)	0.102
C.P.(Yes)	0.040029	(-0.018338, 0.098397)	0.101
F.H.(Yes)	-0.055112	(-0.172698, 0.062473)	0.262
Height	0.802640	(0.706539, 0.898741)	0.000***
(25≤ M.Age <35)	0.035527	(-0.045286, 0.116341)	0.290
M.Age ≤45	0.047127	(-0.053005, 0.147260)	0.185
Pregnancy length (Moderately term)	-0.035037	(-0.176260, 0.106185)	0.432
Pregnancy length (Preterm)	0.165481	(-0.159198, 0.490162)	0.013*
Residency(Urban)	-0.042713	(-0.124909, 0.039483)	0.180
Weight	0.260196	(0.1882976, 0.332094)	0.000***
Height:Weight	-0.247623	(-0.281387, -0.213859)	0.000***
Birth order:3rd:City(Jeddah)	-0.109726	(-0.306868, 0.087414)	0.019*
birth weight:height	0.193901	(0.145237, 0.242564)	0.000***
birth weight:weight	-0.156067	(-0.211721, -0.100412)	0.001**
Pregnancy Length (Preterm):Residency(Urban)	-0.162503	(-0.506012, 0.181006)	0.014*
Height:(25≤ M.Age <35)	-0.160606	(-0.237566, -0.083646)	0.001*
Birth weight:C.P.(Yes)	-0.069193	(-0.130551, -0.007835)	0.064.
birth year:City(Jeddah)	0.107713	(0.031949, 0.183477)	0.014*
C.P.(Yes):height	-0.067283	(-0.127698, -0.006868)	0.07.
Birth weight:Height:Weight	0.086841	(0.061864, 0.111818)	0.020*

***Significant at p-value <0.001,

**Significant at p-value <0.01,

*Significant at p-value <0.05,

^.^Significant at p-value <0.1

### Selection of the significant variables based on ANN models

**Fig 12:**
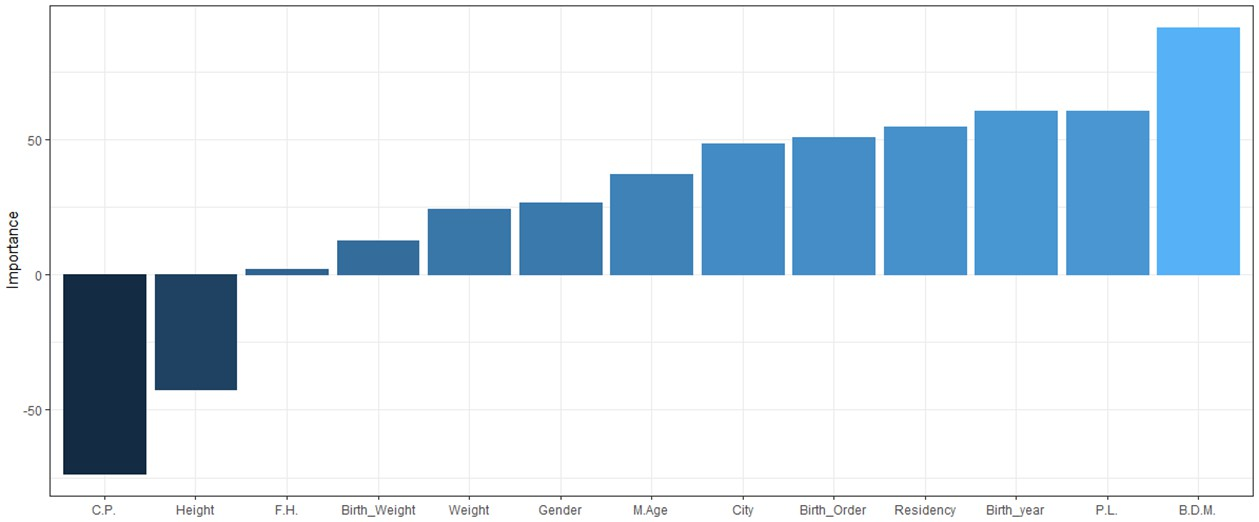
Relative importance of each variable using Olden’s algorithm. C.P.:Consanguineous Parents, P.L.: pregnancy length, F.H.: having a family history of T1D, B.D.M.: Birth delivery mode.

Olden’s algorithm [75] uses the product of raw link weights between the input and the output neuron, and sums the product over all the hidden neurons. A benefit of this method is that the relative contributions of each connection weight in terms of magnitude and sign are retained. This algorithm was used for the ANN model to investigate the relative importance of each variable and is shown in (Fig 12). The figure reveals that some variables have a positive, and some have a negative, relationship with the age at onset of T1D. The results suggest that birth delivery mode has the strongest positive relationship and consanguineous parents has the strongest negative relationship. Similarly, variables that have relative importance close to zero, such as having a family history of T1D does not have any substantial importance for the response variable age at onset.

### Selection of the significant variables based on RF models

**Fig 13:**
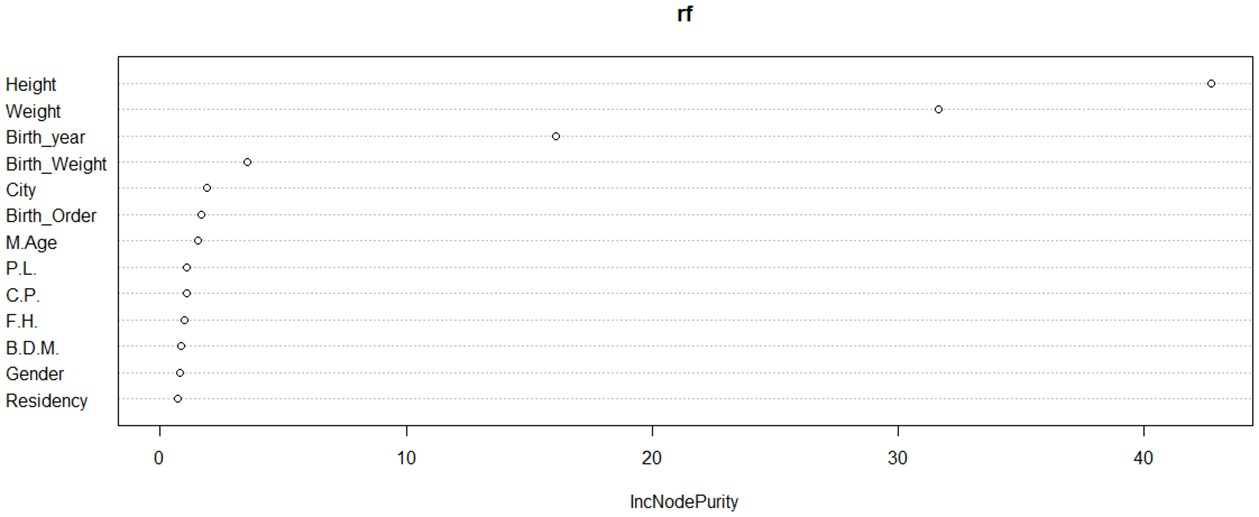
Importance variables based on RF model. C.P.:Consanguineous Parents, P.L.: pregnancy length, F.H.: having a family history of T1D, B.D.M.: Birth delivery mode.

RF was used to identify the important variables that influence the age at onset of T1D (Fig 13). The figure shows that the child’s weight and height at diagnosis, along with the birth year are the most important variables for the response variable followed by birth weight.

## Modeling age at onset of T1D for 5–9 years age group

It has previously been reported that the incidence rate of T1D cases in Saudi Arabia was higher for the age group ≥5 years compared to the age group <5 years [76, 77]. A similar conclusion was made in Italy [21]. This is also observed in our cohort as shown in Figs 1 and 6 where the most common age for children with T1D is between 5 and 9 years. This section utilized MLR, ANN, and RF to model age at onset for this specific age group. The sample size for this age group is 170 children from three different cities of Saudi Arabia. The group has a mean of 6.9 years, standard deviation of 1.4 years, median of 6.9 and mode at 6.1 and 8.1 years. The mean height of this group was (1.23 ± 0.11) metres, the mean weight was (23.2 ± 7.98) kg and the mean birth weight was (2.96 ± 0.68) kg. The median birth year in this group was 2010. Table 11 shows the description of the data for this age group.

**Table 11:** Descriptive statistics for age group of (5–9) years (47.4%).

Variable	No. of cases(%)
City: Al-Ahsa	47(27.6)
Jeddah	77(45.3)
Riyadh	46(27.0)
Gender: Female	93(54.7)
Male	77(45.3)
Consanguineous parents (C.P.): Yes	80(47.1)
No	90(53.0)
Residency: Urban	117(68.8)
Rural	53(31.2)
Family history of type 1 diabetes (F.H.):Yes	74(43.5)
No	96(56.5)
Birth Delivery Mode(B.D.M.): Cesarean section(CS)	53(31.2)
Normal delivery	117(68.8)
Pregnancy Length (P.L.):Full Term	126(74.1)
Moderately Preterm	31(18.2)
Preterm	13(7.6)
Maternal age at child’s birth (M.Age): M.Age <25	31(18.2)
(25≤ M.Age <35)	95(55.9)
M.Age ≤45	44(25.9)
Birth order: 1st	55(32.4)
2nd	29(17.1)
3rd	38(22.3)
4th	48(28.2)

### Multiple linear regression models for 5–9 years age group

MLR models of age at onset of childhood T1D has been developed for this age group. The MLR modeling for the full cohort showed that interactions improved the performance of MLR models, and hence has been examined also in this section as shown in Table 12 with list of the independent and interaction variables in each MLR model. Table 13 illustrates the results of MLR models for this age group without and with interactions between variables. Based on *R*^2^, adjusted *R*^2^, RMSE and MAE, the MLR model M12 using the logarithm of age at onset with interactions between variables was the best model.

**Table 12:** Variables in selected MLR models in age group (5–9).

MLR models	Independent and Interaction Variables
(a) M 10:y	birth Weight, birth year, city, C.P., B.D.M., gender, height, M.Age, P.L., residency, weight, B.D.M.:M.Age, P.L.:residency height:weight, city:C.P., birth year:M.Age, birth weight:gender, P.L.:weight, birth year:C.P., birth weight:city, gender:weight, gender:residency,birth weight:C.P., birth weight:B.D.M.
(a) M 11: sqrt(y)	birth Weight, birth year, city, C.P., B.D.M., gender, height, M.Age, P.L., weight, birth weight:B.D.M., birth year:M.Age, B.D.M.:M.Age, height:P.L., city:C.P., birth year:C.P., birth weight:C.P., birth weight:gender, gender:weight, birth weight:city, C.P.:B.D.M.
(a) M 12: log(y)	birth Weight, birth year, city, C.P., B.D.M., gender, height, M.Age, P.L., weight, height:weight, B.D.M.:M.Age, city:C.P., height:P.L., birth weight:B.D.M., birth year:M.Age, birth year:C.P., birth weight:C.P., C.P.:B.D.M., gender:weight, birth weight:gender, birth weight:city.

C.P.:Consanguineous Parents, P.L.: pregnancy length, B.D.M.: Birth delivery mode, M.Age: Maternal age at child’s birth, (:): interactions between variables.

**Table 13:** MLR models of age at onset of T1D of age group (5–9).

Models without interactions	*R* ^2^	Adj*R*^2^	RMSE	MAE
M 7:lm(y, x’s)	0.44	0.37	1.02	0.83
M 8:lm(sqrt(y), x’s)	0.44	0.37	0.19	0.16
M 9:lm(log(y), x’s)	0.44	0.37	0.15	0.12
Models with interactions	*R* ^2^	Adj*R*^2^	RMSE	MAE
M 10:lm(y, x’s)	0.64	0.55	0.82	0.67
M 11:lm(sqrt(y), x’s)	0.63	0.55	0.16	0.13
M 12:lm(log(y), x’s)	0.64	0.56	0.12	0.10

**Fig 14:**
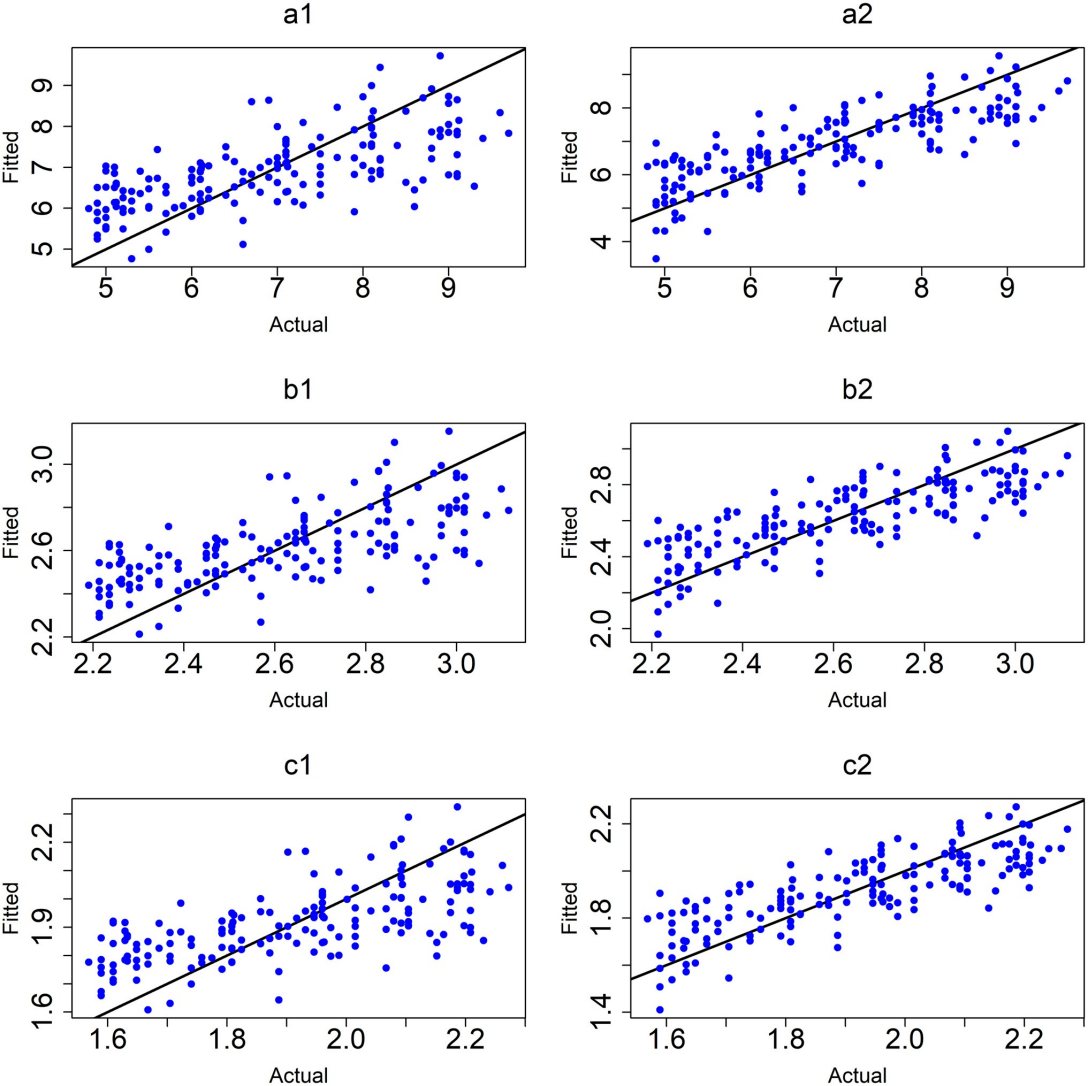
MLR models of age group (5–9). (a1) M 7:y without interactions and (a2)M 10:y with interactions, (b1) M 8:sqrt(y) without interactions and (b2) M 11:sqrt(y) with interactions and (c1) M 9:log(y) without interactions and (c2) M 12:log(y)with interactions.

In Table 13, the MLR models without interactions have *R*^2^ of (0.44) with RMSE (1.02, 0.19 and 0.15) and MAE (0.83, 0.16 and 0.12) for age at onset, square root and logarithm of age at onset respectively. A small improvement in the MLR models was observed by adding interactions. In the models with interactions, MLR models M10-M12, *R*^2^ were (0.64, 0.63, and 0.64), RMSE were (0.82, 0.16 and 0.12) and MAE were (0.67, 0.13 and 0.10) respectively.

(Fig 14) displays the plots of observed values versus fitted values for the MLR models without and with interactions between variables for 5–9 years age group.

From Table 13, the best model of age at onset of T1D was the logarithm model with interactions between variables (c2). However, the MLR for this age group has not performed as well as the MLR for the full cohort.

### Artificial neural network models for 5–9 years age group

ANN models were also utilized to model the age at onset of T1D in the age group (5–9). Data in this age group were randomly divided into two subsets in a ratio of 80% for training to build the ANN models and 20% to use as a testing set to validate the models. The input layer has 13 inputs, two hidden layers were used with 13 neurons and the output layer has one output as with the full cohort. Table 14 shows the results of ANN models in the age group of 5–9 years.

**Table 14:** ANN models of age at onset of T1D of age group (5–9).

Models	Training data			Testing data		
	*R* ^2^	RMSE	MAE	*R* ^2^	RMSE	MAE
A4: y	0.23	2.07	1.72	0.16	2.19	1.85
A5:sqrt(y)	0.13	0.74	0.70	0.12	0.71	0.67
A6:log(y)	0.18	0.47	0.43	0.14	0.49	0.45

**Fig 15:**
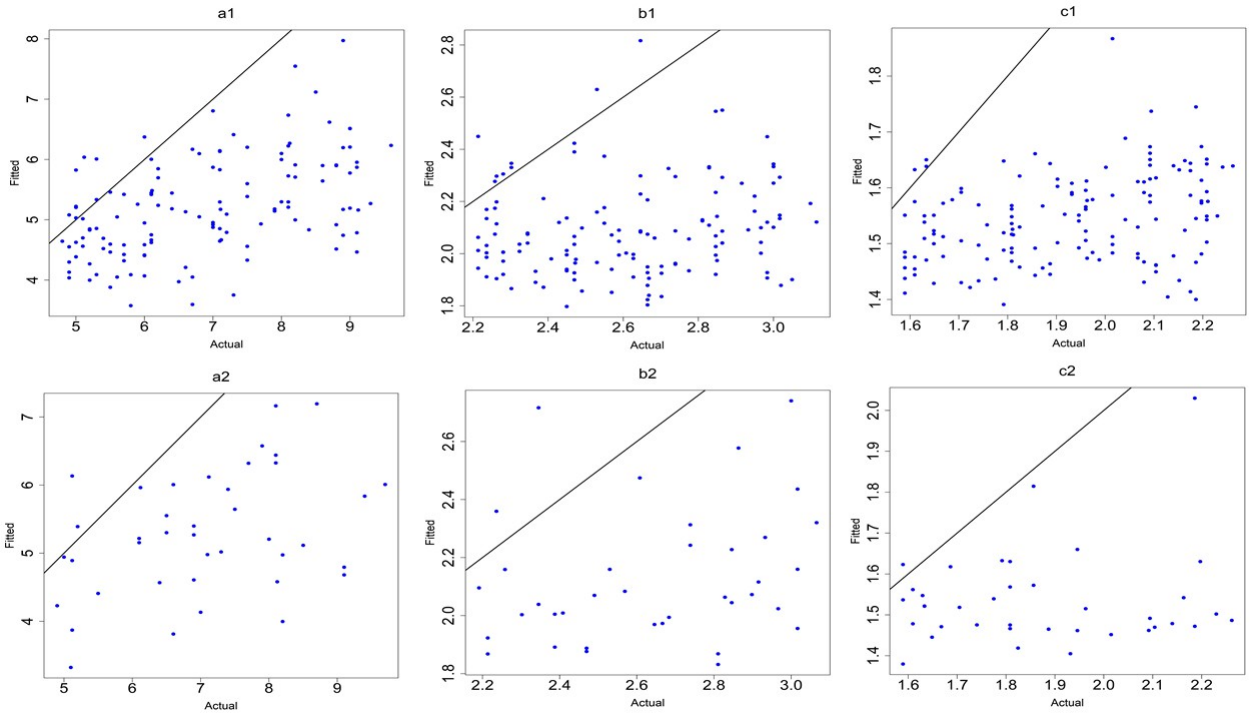
Plots of training and testing data of ANN models for the 5–9 years age group. (a1,a2) y: age at onset, (b1,b2) y: the square root of age at onset, (c1,c2) y: the logarithm of age at onset.

The ANN models for age at onset of T1D have values of *R*^2^ (0.16, 0.12 and 0.14) and RMSE values of (2.19, 0.71 and 0.49) and MAE of (1.85, 0.67 and 0.45) respectively for models A4-A6. The low values of *R*^2^ of ANN models indicates a poor fit to the data for age at onset of T1D in this age group.

(Fig 15) shows the results of the observed values versus predicted values for the ANN models of (5–9) age group.

### Random Forest models for 5–9 years age group

RF models were also used to model the age at onset of T1D in this age group. Data divided into two subsets in a ratio of 80% for training to build the RF models and 20% to use as a testing set to validate the models. Table 15 shows the results of RF models in this age group.

**Table 15:** Random forest models of (5–9) age at onset of T1D.

Models	Training data			Testing data		
	*R* ^2^	RMSE	MAE	*R* ^2^	RMSE	MAE
RF4: y	0.90	0.53	0.42	0.77	0.59	0.51
RF5:sqrt(y)	0.90	0.10	0.08	0.78	0.11	0.09
RF6:log(y)	0.90	0.08	0.06	0.78	0.08	0.07

**Fig 16:**
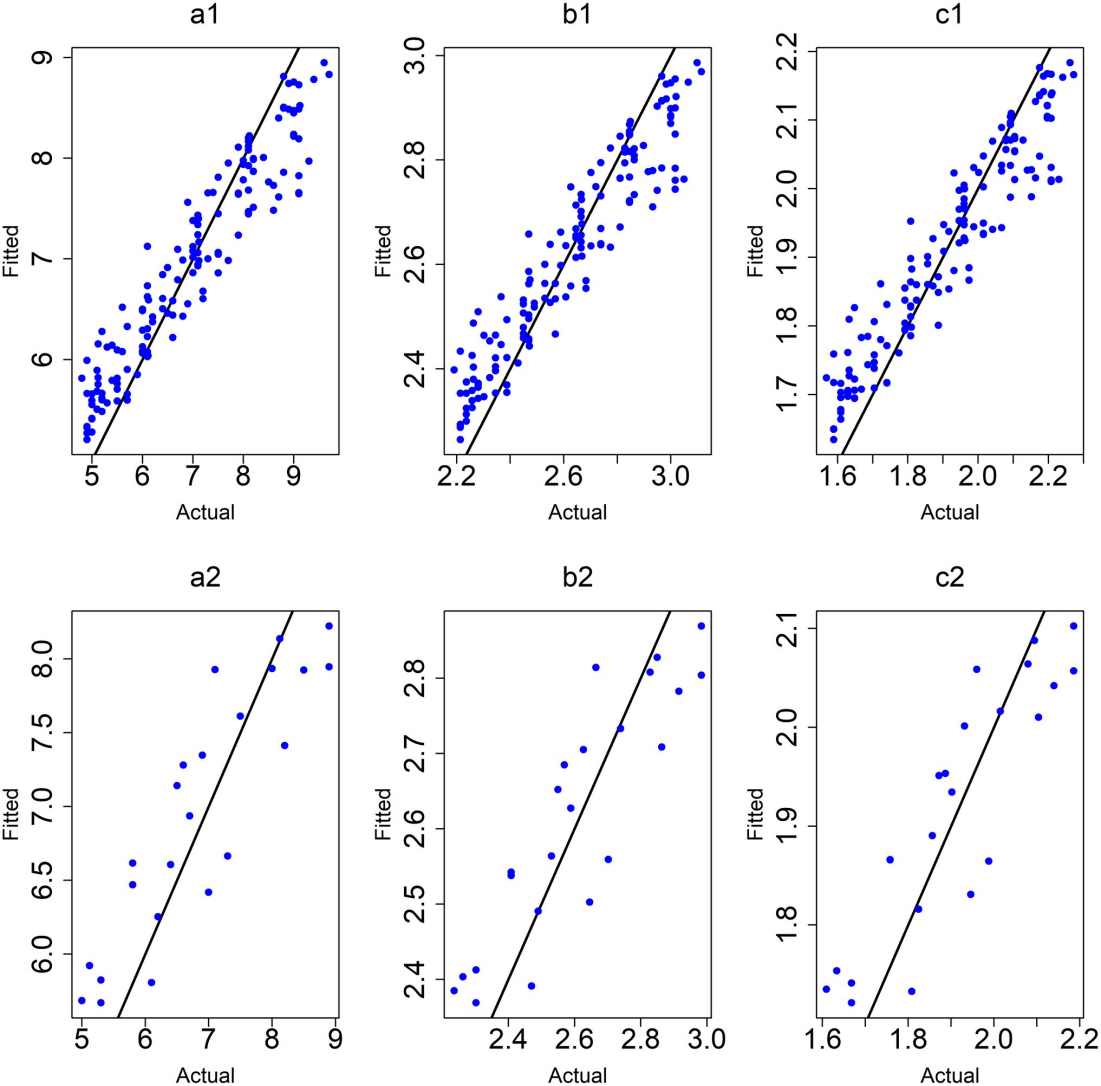
Plots of training and testing data of RF models for the 5–9 years age group. (a1,a2) y: age at onset, (b1,b2) y: the square root of age at onset, (c1,c2) y: the logarithm of age at onset.

In Table 15, the RF models for age at onset of T1D have values of *R*^2^ (0.77, 0.78 and 0.78) and RMSE values of (0.59, 0.11 and 0.08) and MAE of (0.51, 0.09 and 0.07) respectively for models RF4-RF6. Therefore, the best RF model RF6 was again using the logarithm of age at onset. The RF models have the highest values of *R*^2^ compared to MLR and ANN models in this age group.

(Fig 16) shows the results of the observed values versus predicted values for the RF models of (5–9) age group for both training and testing subgroups.

## Validation of models for the 5–9 years age group

This section assesses the efficacy of the selected MLR, ANN and RF models. Data were divided into two subsets of 80% for training and 20% for use as a testing set. The values of *R*^2^, RMSE and MAE were used to assess the efficacy of the models. The results presented in Table 16 indicate that the RF model is the best fit for the data.

**Table 16:** Comparison between MLR, ANN ans RF models of age group(5–9).

Models	*R* ^2^	RMSE	MAE	Accuracy
M 12:(log(y), X’s)	0.64	0.11	0.09	0.99
A4:(y, X’s)	0.16	2.19	1.85	0.76
RF6:(log(y), X’s)	0.78	0.08	0.07	0.99

From Table 16, the RF model has a higher *R*^2^ = 0.78 compared to MLR and ANN models. Also, RMSE and MAE values for RF were 0.08 and 0.07 which are smaller than those of MLR and ANN models. Both MLR and RF models have the highest accuracy of 0.99. These results indicate that RF method outperforms MLR and ANN in describing the age at onset of T1D for age between 5–9 years old by achieving lower RMSE and MAE with a higher *R*^2^.

## Risk factors impacting the age at onset of T1D in (5–9) age group based on MLR, ANN and RF models: Environmental factors and family history of diabetes

Similar to the full cohort, we have used MLR, ANN and RF methods to assess the impact of environmental factors on the age at onset of T1D for the (5–9) age group.

The analysis of the variables that influence the age at onset of T1D in this age group based on the best MLR model together with their corresponding P-value and 95% confidence interval is provided in Table 17 (non-significant interaction variables were excluded for brevity).

**Table 17:** Variables of the MLR model of (5–9) age group.

Variables	*β*	CI	P-value
Intercept	0.000000	(-0.082307, 0.082307)	0.000***
Birth weight	0.110212	(0.057909, 0.162515)	0.434
Birth year	0.167600	(0.098144, 0.237055)	0.256
City(Jeddah)	-0.056171	(-0.130877, 0.018534)	0.551
City(Riyadh)	-0.118340	(-0.198961, -0.037718)	0.194
C.P.(Yes)	-0.340172	(-0.425942, -0.254402)	0.002**
B.D.M.(CS)	-0.274467	(-0.412641, -0.136293)	0.092.
Gender(Male)	-0.161451	(-0.208726, -0.114175)	0.007**
Height	0.477437	(0.410969, 0.543905)	0.000***
(25≤ M.Age <35)	-0.118504	(-0.185424, -0.051583)	0.161
M.Age(≤45)	-0.031857	(-0.111263, 0.0475478)	0.718
Pregnancy length (Moderately term)	0.020058	(-0.045571, 0.085689)	0.755
Pregnancy length (Preterm)	0.109521	(0.015196, 0.203846)	0.08.
Weight	0.521233	(0.453352, 0.589114)	0.000***
Height:Weight	-0.238675	(-0.281045, -0.196304)	0.004*
B.D.M.(CS):(25≤ M.Age <35)	0.242348	(0.093814, 0.390882)	0.097.
Height:Pregnancy length (Preterm)	-0.207138	(-0.322874, -0.091401)	0.003**
Birth Weight:.D.M.(CS)	-0.164170	(-0.210277, -0.118063)	0.029*
Birth year:M.Age(≤45)	-0.238962	(-0.315280, -0.162643)	0.007**
City(Jeddah):C.P.(Yes)	0.377780	(0.272095, 0.483465)	0.000***
Birth year:C.P.(Yes)	-0.246687	(-0.304930, -0.188444)	0.005**
Birth weight:C.P.(Yes)	-0.172882	(-0.216202, -0.129561)	0.041*
Gender(Male):Weight	-0.144286	(-0.206682, -0.081890)	0.057.
Birth weight:Gender(Male)	0.157184	(0.112516, 0.201851)	0.053.
Birth weight:City(Jeddah)	-0.183440	(-0.234028, -0.132852)	0.042*

***Significant at p-value <0.001,

**Significant at p-value <0.0,

*Significant at p-value <0.05,

^.^Significant at p-value <0.1

**Fig 17:**
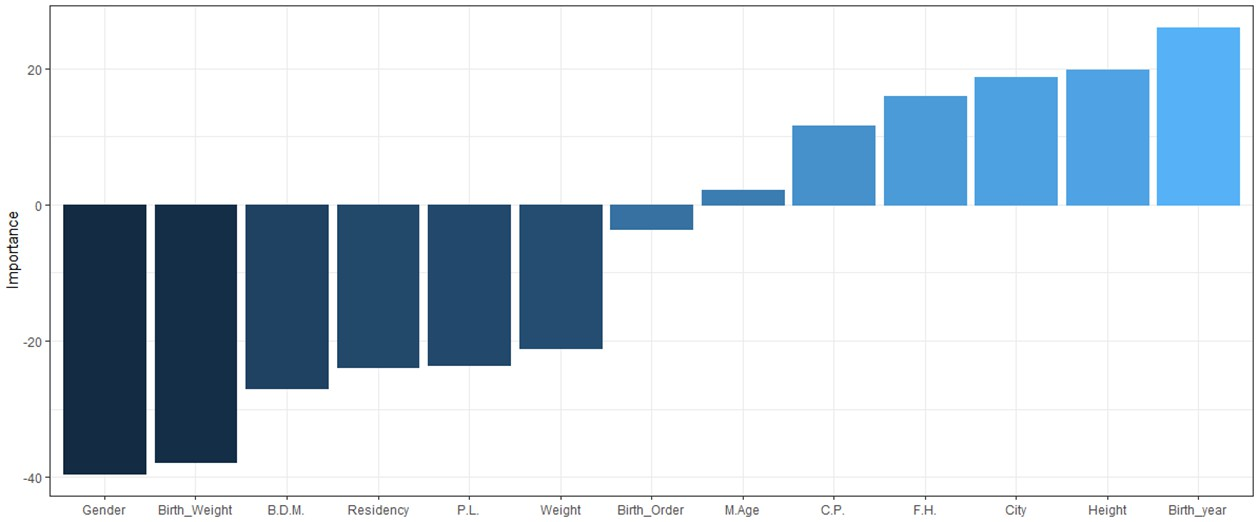
Relative importance of each variable using Olden’s algorithm in (5–9) age group.

Olden’s algorithm is also used in this section to identify the relative importance of variables in the age group (5–9) when utilizing the best performing ANN model (Fig 17). The figure reveals that birth year and gender have the strongest positive and negative relationship respectively with the response variable. Similarly, variables that have relative importance close to zero, such as maternal age at child’s birth does not have any substantial importance for the response variable.

**Fig 18:**
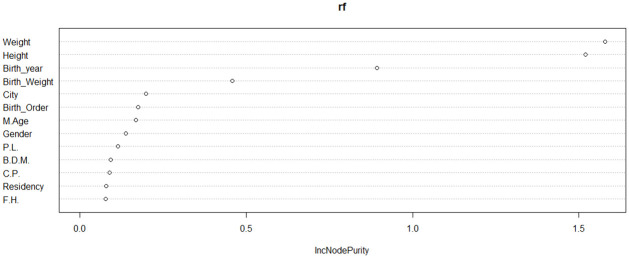
Importance variables based on RF model in (5–9) age group.

Also, RF was used to identify the important variables in the age group (5–9) (Fig 18). The figure indicates that weight and height at diagnosis are the most important variables for the response variable followed by birth year and birth weight.

## Discussion

De-identified data on 359 children with T1D collected from three cities, have been analysed to obtain an insight into the distribution of the age at onset of T1D in Saudi Arabia. The analysis show that there is an overall upward trend in the incidence of T1D in children from 2010 to 2020 with a higher incidence in females. This was also reported for other populations from the Middle East and North Africa (MENA) region [78, 79]. Analyses to identify the factors showing the strongest relationship to the age at onset compared models derived with MLR, ANN and RF. Using the best subset model selection criteria, coefficient of determination, and diagnostic tests of residuals, the most significant independent variables were identified as: city, pregnancy length, consanguineous parents, birth weight, birth year, child’s weight and height at diagnosis. The efficacy of models for predicting the age at onset was assessed using multi-prediction accuracy measures, coefficient of determination (*R*^2^), root mean square error (RMSE) and mean absolute error (MAE). To improve the efficacy of the MLR models, interactions between independent variables were considered. The MLR models were selected based on the step-wise selection criteria of the smallest Akaike’s Information Criteria (AIC).

### Modeling age at onset of T1D

In all MLR, ANN and RF models, different transformations were considered for the age at onset of T1D to find the best model for prediction. The study found the logarithm of age at onset of T1D was the best choice for the dependent variable when using both the MLR and RF methods. The analyses showed that MLR model M3 and RF model RF3 outperformed the ANN model with a higher *R*^2^ = 0.88 and 0.89 and smaller RMSE = (0.22 and 0.21) and MAE = (0.18 and 0.17) respectively.

### The impact of environmental factors and family history of diabetes on the age at onset of T1D

As shown in the analysis in this study, the results support previous findings that the age at onset of T1D can be influenced by environmental factors [17, 21].The result of the MLR model presented here agrees with previous studies conducted in Israel and Australia [17, 80]. It is shown that the birth year (p-value = 0.001) [17] and preterm birth before 37 weeks [17, 80] can influence the age at onset of T1D. In our study, weight and height of child based on RF and MLR results were founded to be associated with age at onset of T1D, in agreement with [24, 25].

In this study, pre-term birth was identified as significant in the MLR model only, whereas an Austrian study found that moderately preterm birth was significant for age at onset of T1D [81]. The MLR model for this data did not show a significant contribution based on family history, in contrast with previous studies [20–22].

### Modeling age at onset of T1D for the age group (5–9)

In this study, we have also focused on creating a model for the (5–9) age group because it was the common age group for onset of T1D in the full data analysis (Fig 6) and as reported previously in the literature [21, 76, 77].

Similar to the full data, the best MLR was the model based on the logarithm of age at onset. The interaction between the variables did not perform well on this subgroup. In the RF models, the logarithm of age at onset was also the best choice for the data. Comparison of the methods based on the values of the *R*^2^, RMSE and MAE achieved shows that RF outperforms the MLR and ANN in describing age at onset of T1D in this cohort.

This study shows that consanguineous parents and gender can influence the age at onset of T1D. Furthermore, it also indicates that birth weight, birth year, weight and height can influence the age at onset of T1D in (5–9) age group.

Utilisation of both traditional statistical multiple linear regression and machine learning approaches should not be regarded as in conflict when the aim is prediction [82]. Although MLR relies on strong assumptions, including the type of error distribution and the additivity of the parameters [82], it has the advantage of being simple to understand the underlying biological relationship. Whereas the results of ANN and RF are often difficult to interpret [82, 83]. ANN and RF analysis provides feature importance, but does not provide complete visibility of the coefficients as linear regression does. However, they may help in understanding the intricate relationships between inputs and determining their impact on the main outcome. They have the flexibility and are free from a priori assumptions. RF has the advantage of a built-in feature selection method, handling many input variables without the need to minimize dimensionality and controls the overfitting by using out-of-bag validation. The other potential disadvantage of machine learning methods revolves around computation complexity. The ANN computation is complex and time-consuming, depending on the type of features used, the number of nodes and layers of the neural network and the number of training data [84]. For large datasets, RF can be computationally intensive as it may require a large number of trees [83]. However, computational time was not an issue in this research due to the fact that the sample size of the data set was not large.

## Conclusions

This study has utilised MLR, ANN and RF to model the age at onset of T1D. The results indicate that the models developed for this data with MLR and RF outperform models using ANN and the best choice for the dependent variable (age at on set) is the the logarithm of age at onset. Results indicated that the selected MLR and RF models can predict the age at onset with a high values of *R*^2^ (0.88 and 0.89) and reasonably small values of RMSE (0.22 and 0.21) and MAE (0.18 and 0.17) for age group of <15 years old. The results also show that the selected RF model with a value of *R*^2^ (0.78) and RMSE and MAE of (0.08 and 0.07) respectively outperforms the other models for age group of (5–9). The low performance for the ANN models is likely a result of the number of observations which subsequent may improve with a larger sample size. As the incidence rate of T1D in children in Saudi Arabia is increasing, it is important for studies to capture the diversity and the culture of the population in Saudi Arabia which is not currently found in the key studies of T1D in children which are from European populations. Hence this study, aimed to address the age at onset of T1D in children in Saudi Arabia utilizing local data and current statistical techniques. The outcomes of this study can effectively aid authorities to identify the risk factors influencing the age at onset of T1D in children to take the appropriate intervention to reduce the impact of delayed T1D diagnosis and potentially slow the incidence rate of T1D in children. This study highlights the need for a national database to further evaluate and improve the model for predictions. To further address the limitations of this study other risk variables, such as maternal weight at childbirth, which has been suggested as a risk factor by Swedish research [27], should also be collected. This is of importance given obesity among females in Saudi Arabia has increased over the past decade [85]. Furthermore, including a larger number of cities in the research would improve both the diversity and increase the sample size, which would consequently provide a more robust model for prediction. In addition, a unified electronic health record between all hospitals in the country will facilitate obtaining pregnancy variables and birth characteristics when a mother’s pregnancy is followed up at hospitals other than the one where gives birth.

## Supporting information

S1 Table Summarise of the literature review. (PDF)

S2 Table ANOVA results comparing age of onset of T1D between gender and cities. (PDF)
