# Supplementary material for: Predicting age at onset of type 1 diabetes in children using regression, artificial neural network and Random Forest: A case study in Saudi Arabia
Source: PLoS One. 2022 Feb 28;17(2):e0264118. doi: 10.1371/journal.pone.0264118 (PMC8884498; doi:10.1371/journal.pone.0264118)
Supplement: S1 Table — (PDF) [file pone.0264118.s001.pdf]

S1 Table. Summarise of the literature review.

Authors<br><br>Year	Title	Methodology & Design	Aim	Setting & sample size	Key findings	Limitations
Adar A, Shalitin S, Eyal O, Loewenthal N, Pinhas-Hamiel O, Zuckerman Levin N, Dally-Gottfried O, Landau Z, Zung A, Levy-Khademi F, Zangen D.<br><br>2020[17]	Birth during the moderate weather seasons is associated with early onset of type 1 diabetes in the Mediterranean area	T-test<br>&<br>Chi-square<br>&<br>multivariate analysis (logistic regression)<br><br>A cross-sectional review of all medical records	To assess the association of seasonal and perinatal parameters with early age of type 1 diabetes (T1D) onset.	Israel<br><br>1571	birth season, birth year, Preterm (<37weeks), Birth weight, gestational age size were associated with earlier T1D.<br>No difference was found in gender, ethnicity, history of autoimmune disease in family or T1D.	A cross-sectional study. There is no data regarding environmental pollution as toxins.<br>The seasonal variations and environmental effects may not be generalizable to other areas in the world with different climates and environmental exposures
Kuchlbauer V, Vogel M, Gausche R, Kapellen T, Rothe U, Vogel C, Pfäffle R, Kiess W.<br>2014 [18]	High birth weights but not excessive weight gain prior to manifestation are related to earlier onset of diabetes in childhood: 'accelerator hypothesis' revisited	T-test<br>&<br>ANOVA<br>&<br>linear mixed effect model<br><br>A follow-up analysis	To test Wilkin's 'accelerator hypothesis': whether excessive weight gain accelerates the onset of type 1 diabetes.	Germany<br><br>1117	No effect of weight gain in the years before diagnosis on age at diagnosis.<br>However, children with diabetes had consistently higher BMI SDS in trend analysis<br>Children with diabetes had a significantly higher birth weight.	
Hsin-Yu Lee, Chin-Li Lu, Hua-Fen Chen, Hui-Fang Su, Chung-Yi Li<br><br>2015 [19]	Perinatal and childhood risk factors for early-onset type 1 diabetes: a population-based case-control study in Taiwan	conditional logistic regression<br><br>A nested case-control study	To investigate the relationships between various perinatal and childhood risk factors and T1D incidence in young children (<10 years)	Taiwan<br><br><br>632 Cases<br>6320 control	Children's prior history of infection, maternal age at delivery of 25–29 years, caesarean section, gestational diabetes, pre-eclampsia, mother with history of T1D, Fathers age 30 and older and a father with family history of both T1D and T2D were risk factors of early T1D in children.	This study was unable to comprehensively take all potential genetic,environmental,perinatal/neonatal risk factors into account because of limited information available from claim data.<br>the infections investigated included only those infections leading to hospitalization and excluded most childhood infections.<br>Due to unavailability of all medical claims for parents, this study analysed only the mothers' inpatient claims

					However, preterm, low birth, residing in high population density areas and maternal age at delivery (≥35 years) were not significant risk factors.	on pre-eclampsia, gestational diabetes, history of admission for infection during pregnancy, which may have under-reported the prevalence of these maternal conditions.
E.J.K. Wadsworth, J.P.H. Shield, L.P. Hunt, J.D. Baum<br><br>1997 [20]	A Case-control Study of Environmental Factors Associated with Diabetes in the Under 5s	Conditional logistic regression<br><br>Case-control Study	To investigate whether the increase in incidence in this age group was continuing,26 and to clarify the strength of association of certain environmental factors in the development of childhood-onset diabetes in the under 5s,	UK<br><br>218	Paternal T1D is associated with increased risk, and higher birth order and paternal age greater than 25 years with decreased risk of diabetes.	
Altobelli E, Petrocelli R, Verrotti A, Chiarelli F, Marziliano C.<br><br>2016[21]	Genetic and environmental factors affect the onset of type 1 diabetes mellitus	T test<br>&<br>two-way ANOVA	To investigates T1DM time trends from 1989 to 2008 and tries to establish whether breast/bottle feeding, a family history of diabetes, and childhood infectious diseases influence age at onset.	Italy<br><br>461	Early T1D onset was related to mixed feeding, a family history of T1D whereas multiple bacterial infection s delayed age at onset.<br>History of T2D was not found to affect age at onset.	The prevalence data of bacterial infections could be underestimated. As regards feeding, since the study considered only feeding type, not the time of introduction of solid food, there may be an effect due to the latter factor that the analysis does not consider.
Małachowska B, Baranowska-Jaźwiecka A, Hogendorf A, Szadkowska A, Fendler W, Młynarski W. 2012[22]	Unequal contribution of familial factors to autoimmunity and clinical course of childhood diabetes	Mann-Whitney's U test<br>&<br>Chi-Square test	This study was performed in order to describe detailed familial history of DM in patients and to evaluate the impact of it on the natural course of childhood DM	Poland<br><br>989	Having siblings with DM was linked to early onset.<br>Maternal diabetes was significantly associated with diabetes onset at an older age.	Long observation period (difference between the youngest and the oldest patients reached 20 years) could have decreased the homogeneity of the population.
Rubio-Cabezas O, Patch AM, Minton JA, Flanagan SE, Edghill EL, Hussain K, Balafrej A, Deeb	Wolcott-Rallison Syndrome Is the Most Common Genetic Cause of Permanent Neonatal Diabetes	χ2 test	To identify patients with WRS before any other abnormalities apart from diabetes are present and study the overall	UK<br><br>34	Wolcott-Rallison syndrome is the most common cause of permanent neonatal diabetes mellitus in	

A, Buchanan CR, Jefferson IG, Mutair A. 2009 [23]	in Consanguineous Families		frequency of WRS among patients with permanent neonatal diabetes.		consanguineous pedigrees.	
Betts P, Mulligan J, Ward P, Smith B, Wilkin T. 2005 [24]	Increasing body weight predicts the earlier onset of insulin-dependent diabetes in childhood: testing the 'accelerator hypothesis' (2)	Case-control design.	To examine recent increase in body mass of children, with associated visceral fat accumulation, possibly the accelerator accounting for the earlier onset and rising incidence of Type 1 diabetes in young people.	England 168 Cases 254 control	There was also a tendency for children who were younger at diagnosis to be taller and heavier	There is no full pre-onset measurements of height and weight, and the waist measurements are both incomplete and all of them post-onset.
Knerr I, Wolf J, Reinehr T, Stachow R, Grabert M, Schober E et al. 2005[25]	The 'accelerator hypothesis': relationship between weight, height, body mass index and age at diagnosis in a large cohort of 9,248 German and Austrian children with type 1 diabetes mellitus	Kruskal–Wallis test & Wilcoxon signed rank test Case-control	To explore the relationships of body weight, height and BMI with onset of type 1 diabetes in a large cohort of Caucasoid patients in whom diabetes became manifest between 1990 and 2003, and to investigate both time courses and age-related effects to test the accelerator hypothesis.	Germany & Austria 9,248 Cases 34,422 controls	Weight and BMI were significantly higher in patients at diagnosis than in controls Patients at diagnosis were significantly taller than controls	
Evertsen J, Alemzadeh R, Wang X. 2009[26]	Increasing incidence of pediatric type 1 diabetes mellitus in Southeastern Wisconsin: relationship with body weight at diagnosis.	logistic multivariate regression	To determine the changing burden of T1DM in specific age cohorts in relationship to BMI and body weight at diagnosis in Southeastern Wisconsin.	Wisconsin 1618	The youngest age groups seem to also be the heaviest	
Lindell N, Carlsson A, Josefsson A, Samuelsson U. 2018 [27]	Maternal obesity as a risk factor for early childhood type 1 diabetes: a nationwide, prospective, population-based case–control study.	χ^2 test & multiple logistic regression Case-control study	To investigate the influence of maternal BMI and gestational weight gain on the subsequent risk of childhood type 1 diabetes.	Swede 3231 Cases 12,948 control	Maternal obesity also correlated with early onset (0–4 years) of type 1 diabetes in the offspring.	
Al-Ghamdi AH, Fureeh AA, Alghamdi JA, Alkuraimi WA,	High prevalence of vitamin D deficiency among Saudi children	T-test. & Chi-square	To evaluate the prevalence of vitamin D deficiency among	Saudi Arabia (Albaha)	Saudi children and adolescent with T1DM in Albaha region	Small sample size and one region

Alomar FF, Alzahrani FA, Alzahrani RA, Alzahrani AA, Alzahrani SA, Alghamdi AM. 2017 [28]	and adolescents with type 1 diabetes In Albaha region, Saudi Arabia	cross-sectional study	Saudi children and adolescents with T1DM in Albaha region, southwestern of Saudi Arabia.	117 (Aged 0-19 years old)	have an alarmingly low vitamin D status.	
Nouf A. ALkharashi<br><br>2019 [29]	Estimation of vitamin D deficiency prevalence among Saudi children in Armed Forces Hospital and Riyadh Care Hospital in Riyadh, Kingdom of Saudi Arabia and its relation to type 1 diabetes mellitus	T-test.<br><br><br>A cross-sectional single centre	To evaluate the prevalence of vitamin D deficiency among a group of Saudi children with type 1 diabetes mellitus (T1DM)	Saudi Arabia (Riyadh)<br><br><br>100 (aged 2-12 years old)	There is a concern that growing children with low vitamin D may be at higher risk for T1DM.	small sample size and cross-sectional-based study cannot suggest any cause of vitamin D deficiency and limits the application of our results to the Saudi population
