# Supplementary material for: Predicting age at onset of type 1 diabetes in children using regression, artificial neural network and Random Forest: A case study in Saudi Arabia
Source: PLoS One. 2022 Feb 28;17(2):e0264118. doi: 10.1371/journal.pone.0264118 (PMC8884498; doi:10.1371/journal.pone.0264118)
Supplement: S2 Table — (PDF) [file pone.0264118.s002.pdf]

S2 Table. ANOVA results comparing age of onset of T1D between gender and cities.

Gender:City	Mean Difference	CI	P-value
Male:AlAhsa-Female:AlAhsa	-0.627709	(-2.594115,1.338695)	0.942
Female:Jeddah-Female:AlAhsa	0.017561	(-1.515431,1.550554)	1.000
Male:Jeddah-Female:AlAhsa	0.582433	(-1.035247,2.200114)	0.907
Female:Riyadh-Female:AlAhsa	-0.273018	(-1.976528,1.430492)	0.997
Male:Riyadh-Female:AlAhsa	-1.436690	(-3.268477,0.395096)	0.218
Female:Jeddah-Male:AlAhsa	0.645271	(-1.220942,2.511484)	0.920
Male:Jeddah-Male:AlAhsa	1.210142	(-0.726239,3.146525)	0.473
Female:Riyadh-Male:AlAhsa	0.354691	(-1.653947,2.363331)	0.995
Male:Riyadh-Male:AlAhsa	-0.808980	(-2.927501,1.309539)	0.883
Male:Jeddah-Female:Jeddah	0.564871	(-0.929415,2.059158)	0.887
Female:Riyadh-Female:Jeddah	-0.290579	(-1.877384,1.296225)	0.995
Male:Riyadh-Female:Jeddah	-1.454251	(-3.178040,0.269536)	0.153
Female:Riyadh-Male:Jeddah	-0.855451	(-2.524215,0.813312)	0.684
Male:Riyadh-Male:Jeddah	-2.019123	(-3.818642,-0.21960)	0.017
Male:Riyadh-Female:Riyadh	-1.163672	(-3.040724,0.713380)	0.482
